# 1,3-propanediol production by *Klebsiella pneumoniae* ∆*dhaM*∆*ptsG*∆*glpK* using glucose and glycerol as co-substrates

**DOI:** 10.1186/s12934-025-02896-6

**Published:** 2025-12-15

**Authors:** Shaoqi Sun, Weiyan Jiang, Yaoyu Cai, Wenqi Wang, Xinjie Bian, Taiyu Liu, Marina Tišma, Dexin Wang, Jian Hao

**Affiliations:** 1 Lab of Biorefinery, Shanghai Advanced Research Institute, Chinese Academy of Sciences, No. 99 Haike Road, Pudong, Shanghai, 201210 People’s Republic of China; 2 Faculty of Food Technology Osijek, Josip Juraj Strossmayer University of Osijek, Franje Kuhača 18, Osijek, HR-31000 Croatia; 3 University of Chinese Academy of Sciences, Beijing, 100049 PR China

**Keywords:** 1,3-propanediol, Glycerol, Glucose, Klebsiella pneumoniae

## Abstract

**Background:**

1,3-Propanediol (1,3-PDO) is a bulk chemical that can be produced by *Klebsiella pneumoniae* using glycerol as a substrate. In the 1,3-PDO synthesis pathway, part of the glycerol is oxidised to maintain intracellular NADH balance. Consequently, the theoretical maximum yield of 1,3-PDO from glycerol was lower than 1 mol/mol.

**Results:**

In this study, engineered *K. pneumoniae* strains were constructed to direct all glycerol toward 1,3-PDO synthesis, with NADH being supplied through the catabolism of glucose. However, glycerol utilisation was inhibited in the presence of glucose. To alleviate this carbon catabolite repression (CCR), *ptsG* and *crr* were individually knocked out. The *dha* pathway is responsible for 1,3-PDO synthesis. Key genes in the oxidation branch of this pathway, including *dhaK*,* dhaL*,* dhaD*, and *gldA*, were knocked out to block this pathway. However, the expression of the *dha* operon was impaired in these strains, resulting in low 1,3-PDO production. In contrast, knocking out *dhaM*, which encodes a subunit of dihydroxyacetone kinase II, effectively blocked the glycerol oxidation pathway while maintaining the activity of the *dha* operon. Additionally, *glpK* was knocked out to block the *sn*-glycerol-3-phosphate formation from glycerol. Glucose to glycerol with the ratio of 0.5:1 mol/mol was the optimal value for 1,3-PDO production by *K. pneumoniae* ∆*dhaM*∆*ptsG*∆*glpK*, leading to a balance of NADH generation and consumption. Microaerobic conditions were favourable for 1,3-PDO production than anaerobic or aerobic conditions. In fed-batch fermentations, this strain produced 58.6 g/L of 1,3-PDO after 70 h, achieving a yield of 0.93 mol/mol glycerol, 2 mol/mol glucose, 0.63 mol/mol substrate.

**Conclusions:**

A highly efficient 1,3-PDO production technology that using glycerol and glucose as co-substrates was established.

**Graphical abstract:**

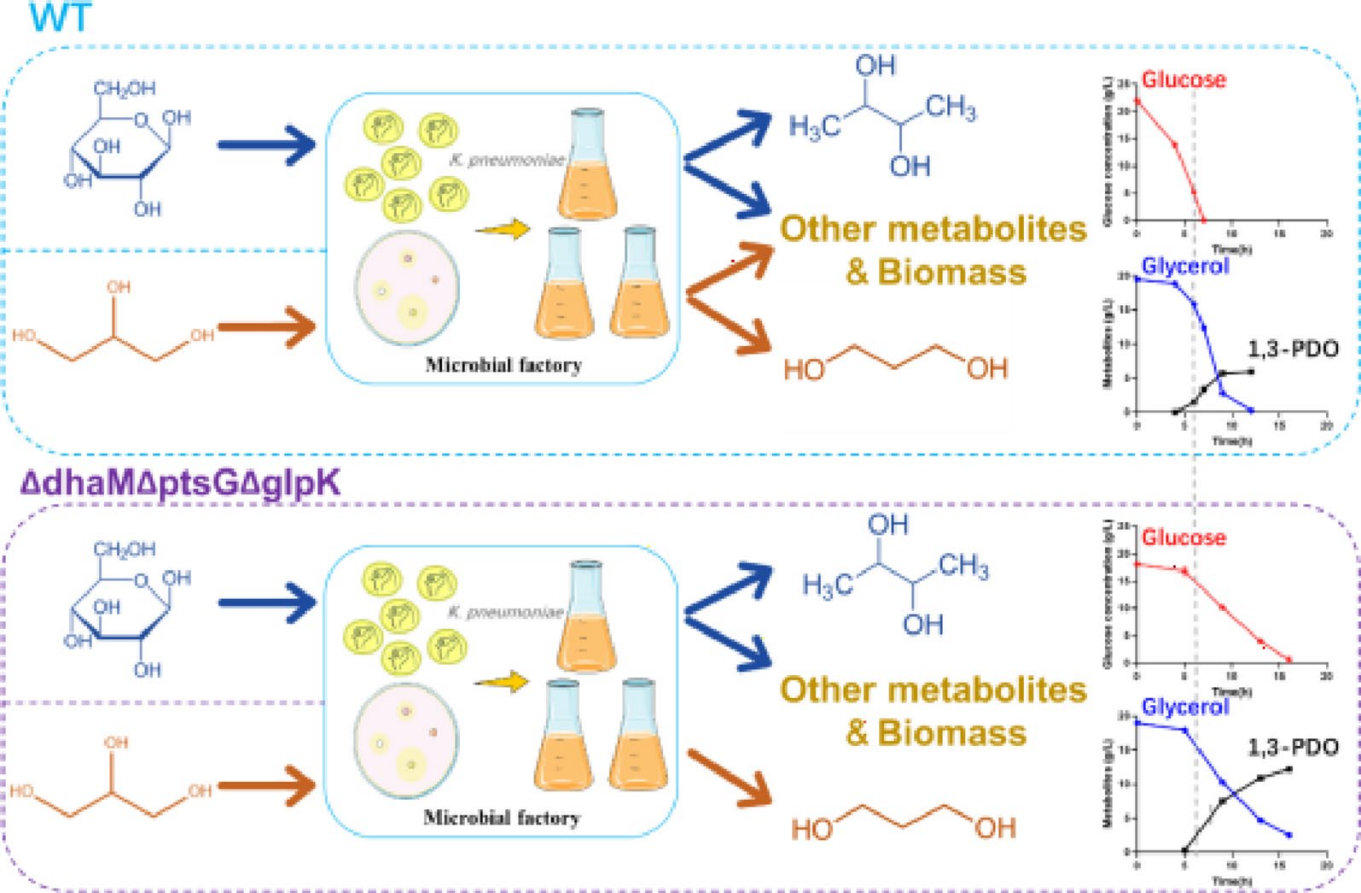

**Supplementary Information:**

The online version contains supplementary material available at 10.1186/s12934-025-02896-6.

## Background

Biodiesel is a renewable alternative energy source valued for its excellent fuel properties, including biodegradability, non-toxicity, and carbon neutrality [1]. Glycerol is the primary by-product in the biodiesel production process, accounting for approximately 10% of the product [2]. 1,3-Propanediol (1,3-PDO), which contains two hydroxyl groups, is an important platform chemical. It serves as a solvent in the cosmetic industry [3]. It is a precursor for synthesising polyester and other polymers [4]. Poly-trimethylene terephthalate (PTT), which was synthesised from 1,3-PDO and terephthalic acid, has outstanding market prospects due to its excellent performance in the textile industry [5]. The market demand for 1,3-PDO is expected to expand with its increasing application. Currently, the commercial 1,3-PDO production has two routes. One route uses glycerol as the feedstock and *Klebsiella pneumoniae* as the producer [4]. The other route uses glucose as the feedstock and uses engineered *Escherichia coli* or *Corynebacterium glutamicum* as producers [6].

*K. pneumoniae* has the characteristics of quick growth and is less susceptible to contamination by other microorganisms during cultivation. Owing to its clear genetic background and metabolic characteristics, *K. pneumoniae* has been used as a chassis for cell factories. Numerous chemicals including 1,2-propanediol [7], isobutanol [8], acetoin [9], dihydroxyacetone [10], 2-ketoisovalerate [8], valine [11], 2,3-butanediol [12], 2-hydroxyislvalerate [13], 2-ketogluconic acid [14], xylonic acid [15], 2,3-dihydroxyisovalerate [16] and gluconic acid [17] were produced by engineered *K. pneumoniae* strains.

**Fig. 1:**
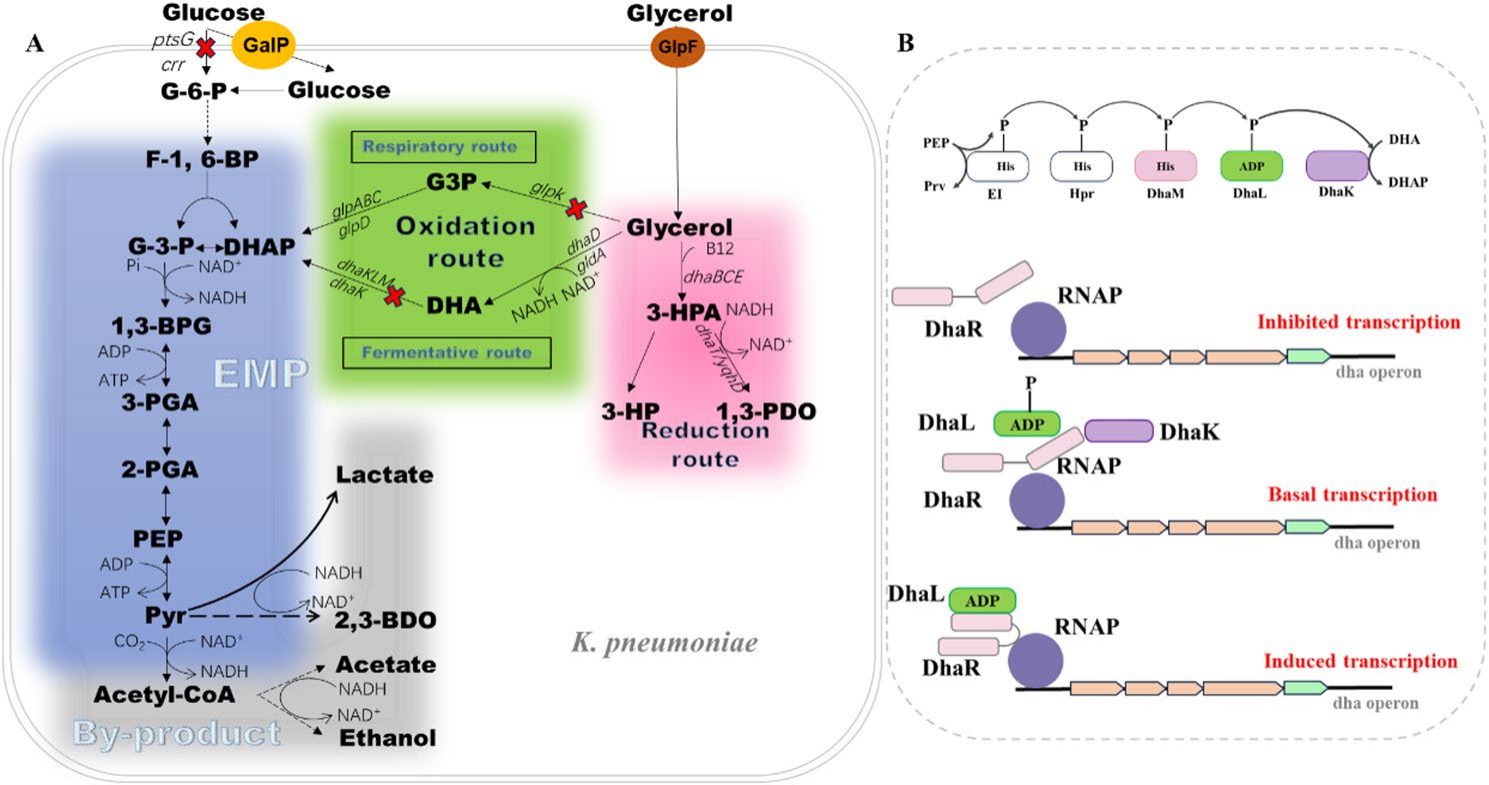
(A) Schematic diagram of the 1,3-PDO synthesis pathway and related pathways in *K. pneumoniae*. Solid lines represent a single-step reaction, while dashed lines indicate multi-step enzymatic reactions. Red crosses indicate knocked-out genes. Blue background: Glycolysis pathway, Green background: Glycerol oxidation route, Pink background: Glycerol reduction route, Grey background: By-product formation pathway. (B) Model of transcription control by the Dha kinase. Boldface letters indicate metabolites. G-6-P, glucose-6-phosphate; F-1,6-BP, fructose 1,6-bisphosphate; G-3-P, glyceraldehyde 3-phosphate; DHAP, dihydroxyacetone phosphate; 1,3-BPG, 1,3-bisphospho-D-glycerate; 3-PGA, 3-phosphoglycerate; 2-PGA, glycerate 2-phosphate; PEP, phosphoenolpyruvate; Pyr, pyruvate; 2,3-BDO, 2,3-butanediol; G3P, glycerol 3-phosphate; DHA, dihydroxyacetone; 3-HPA, 3-hydroxypropanal; 3-HP, 3-hydroxypropionic acid; 1,3-PDO, 1,3-propanediol. GlpF, glycerol transporter; GalP, galactose-proton symporter; RNAP, RNA polymerase; EI, Hpr, phosphoenolpyruvate-dependent phosphotransferase system (PTS) protein; Italicized letters indicate metabolic enzymes: *ptsG*, EIICB; *crr*, EIIA; *glpABC*/*glpD*, glycerol-3-phosphate dehydrogenase; *glpK*, glycerol kinase; *dhaK1*, dihydroxyacetone kinase I; *dhaKLM*, dihydroxyacetone kinase II; *dhaD*/*gldA*, glycerol dehydrogenase; *dhaBCE*, glycerol dehydrogenase; *dhaT*/*yqhD*, 1,3-PDO oxidoreductase

1,3-PDO synthesis from glycerol via the *dha* pathway, which is a branched pathway (Fig. 1A). In the reduction branch, glycerol is dehydrated to form 3-hydroxypropionaldehyde (3-HPA) catalysed by a vitamin B_12_-dependent glycerol dehydratase (*dhaBCE*). 3-HPA is subsequently reduced to 1,3-PDO by a NADH-dependent 1,3-PDO oxidoreductase (*dhaT*). In the oxidation branch of the *dha* pathway, glycerol is oxidised to dihydroxyacetone by glycerol dehydrogenase (*dhaD*, *gldA*) using NAD^+^ as an electron acceptor. Dihydroxyacetone is phosphorylated to form dihydroxyacetone phosphate. *dhaKLM* encodes a PEP-dependent dihydroxyacetone kinase Ⅱ. DhaR is a transcription activator from the family of enhancer binding proteins. DhaK binds to the sensing domain of DhaR (Fig. 1B) and thereby keeps DhaR in a transcription inactive state. Dephosphorylated DhaL displaces DhaK and activates DhaR. DhaM rephosphorylates DhaL to inhibit the transcription of *dha* operon. Besides, there is an ATP-dependent dihydroxyacetone kinase Ⅰ in the *dha* pathway, which is encoded by *dhaK1* [18]. Dihydroxyacetone phosphate obtained from glycerol oxidation is channeled into glycolytic pathway. NADH, energy and building blocks of the cell are generated in glycolysis and downstream pathways. Ethanol, 2,3-butanediol, acetate, succinate, and lactate are the main metabolic by-products derived from glycerol oxidation.

Besides the oxidation branch of *dha* pathway, there is a *glp* pathway for glycerol oxidation [19]. In this pathway, glycerol is phosphorylated to *sn*-glycerol-3-phosphate by glycerol kinase (*glpK*), then *sn*-glycerol-3-phosphate is oxidised to dihydroxyacetone phosphate by glycerol-3-phosphate dehydrogenase (*glpD*, *glpABC*).

1,3-PDO synthesis through the reduction branch of *dha* pathway consumes NADH. Whereas NADH is generated in the glycerol oxidation pathways. This coupling maintains an intracellular NADH balance [20], which limits the yield of 1,3-PDO from glycerol. The theoretical maximum value has been reported to be 0.67–0.875 mol/mol, while the experimental yields typically range from 0.35 to 0.65 mol/mol [19].

To improve the yield of 1,3-PDO synthesis from glycerol, glucose was introduced as a co-substrate. In the co-substrate process, NADH, energy and building blocks of the cell were supported through glucose catabolism, allowing all glycerol to be channeled toward 1,3-PDO synthesis. The theoretical maximum yield of glycerol to 1,3-PDO reached 1.00 mol/mol [21]. A yield of 0.988 mol/mol glycerol and a final 1,3-PDO titer of 68.32 g/L were achieved by *Lactobacillus reuteri* using glucose and glycerol as co-substrates, and a final 1,3-PDO titer of 68.32 g/L was obtained [22]. Besides glucose, lignocellulosic hydrolysate was used as a co-substrate for 1,3-PDO production with *Clostridium diolis* [23].

It has been reported that growing *K. pneumoniae* with glucose as the primary carbon source and glycerol as a co-substrate can enhance the conversion efficiency of glycerol to 1,3-PDO. However, the yields remained low, with 0.53 mol/mol glycerol and 0.48 mol/mol substrate (glucose and glycerol) [24]. It has been found that the activities of key enzymes responsible for 1,3-PDO synthesis were significantly reduced in the co-substrate process [25]. This is a major challenge that needs to be overcome in the co-substrates process.

In this work, engineered *K. pneumoniae* strains were constructed to direct all glycerol used for 1,3-PDO synthesis. Shake flasks were used to evaluate the performance of strains. Then, bioreactors were used for the cultivation parameters optimisation. Finally, high yield and final titer of 1,3-PDO were obtained in fed-batch fermentations.

## Results

### Alleviation of carbon catabolite repression (CCR)

Many natural bacteria can produce 1,3-PDO from glycerol. *K. pneumoniae* is a prominent producer due to its high substrate conversion ratio and high 1,3-PDO titers, and its production technology has been industrialised. *K. pneumoniae* CGMCC 1.6366 was isolated from the rhizosphere, and the wild-type strain does not contain any plasmids. This strain was an efficient 1,3-PDO producer [26]. Glucose can be used as a substrate by *K. pneumoniae* for 2,3-butanediol and other chemicals production, but not for 1,3-PDO.

**Fig. 2:**
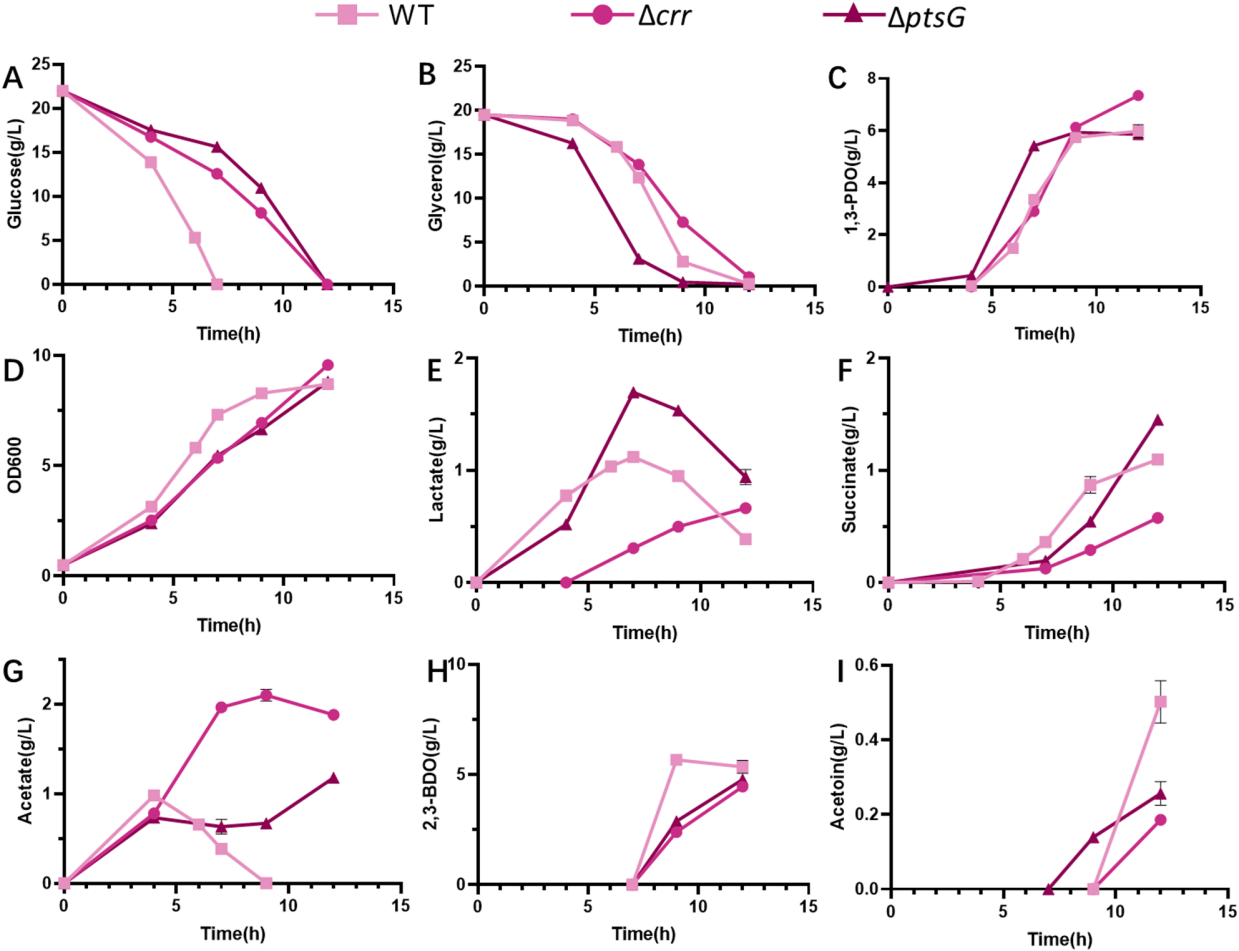
Shake flask experiments: Cell growth, substrates (glucose and glycerol) depletion, product (1,3-PDO) and by-products (lactate, succinate, acetate, 2,3-BDO and acetoin) formation during *K. pneumoniae* (Kp-WT), *K. pneumoniae* ∆*ptsG* (Kp ∆*ptsG*), and *K. pneumoniae* ∆*crr* (Kp ∆*crr*) cultures. Values are presented as the mean ± standard error (*n* = 3)

Here, the wild-type *K. pneumoniae* was cultured in shake flasks for 1,3-PDO production, with a medium containing 22 g/L of glucose and 20 g/L of glycerol. The fermentation results are presented in Fig. 2. As shown in Fig. 2, glucose was exhausted after 7 h of cultivation. Glycerol was not consumed by the cells during the first 4 h. 7.1 g/L of glycerol was consumed after 7 h of cultivation. After 13 h of cultivation, glycerol was exhausted, and 6.0 g/L of 1,3-PDO was produced in the broth. The yield of 1,3-PDO from glycerol was 0.37 mol/mol. The final cell density was 8.7 OD units. Besides 1,3-PDO, lactate, succinate, acetate, 2,3-butanediol (2,3-BDO), and acetoin were produced as by-products. These results indicated that the utilisation of glycerol was suppressed when glucose was present in the medium. If glucose and other carbon sources are present in the culture medium, bacteria prefer to utilise glucose. Until glucose is exhausted, other carbon sources are then used by the cell. This rule is carbon catabolite repression (CCR). As expected, carbon source utilisation by *K. pneumoniae* is subject to CCR.

EIIA^Glc^ (*crr*) and EIICB (*ptsG*) are components of the glucose-specific phosphoenolpyruvate-dependent transferase system (PTS). It has been reported that deletion of *crr* or *ptsG* eliminates CCR in bacteria, including *K. pneumoniae* [24, 27]. Here, *crr* and *ptsG* were knocked out individually to obtain *K. pneumoniae* ∆*ptsG* and *K. pneumoniae* ∆*crr*. These two strains were cultured in shake flasks, and the results are presented in Fig. 2.

Glucose consumption rates of *K. pneumoniae* ∆*ptsG* and *K. pneumoniae* ∆*crr* were slower than those of the wild-type strain. It took 12 h for the two strains to consume the 22 g/L of glucose. Unlike the wild-type strain, glycerol was consumed simultaneously with glucose by the two strains. The glucose consumption rate of *K. pneumoniae* ∆*crr* was faster than that of *K. pneumoniae* ∆*ptsG*. Whereas, *K. pneumoniae* ∆*ptsG* exhibited a fast glycerol consumption rate. 7.3 g/L and 5.8 g/L of 1,3-PDO were produced by the two strains, and the yields were 0.48 and 0.36 mol/mol glycerol. Compared to the wild-type strain, *K. pneumoniae* ∆*crr* produced more 1,3-PDO. Deletion of *ptsG* did not affect the final 1,3-PDO titer or yield, but it enhanced productivity. The cell growth of wild-type *K. pneumoniae* was faster than that of the two engineered strains. After 9 h, the cell density of *K. pneumoniae* ∆*ptsG* and *K. pneumoniae* ∆*crr* were 6.7 and 7.0 OD units, respectively. After 12 h, the cell densities of the two strains reached 8.8 and 9.6 OD units. This suggests that the knockout of *ptsG* or *crr* impaired the cell growth.

Lactate, succinate, acetate, 2,3-BDO, and acetoin were byproducts of these processes. Lactate produced by the wild-type strain, *K. pneumoniae* ∆*ptsG* and *K. pneumoniae* ∆*crr* were 0.4, 0.9, and 0.7 g/L, respectively. Succinate titers were 1.1, 1.5 and 0.5 g/L. Acetate was produced by the wild-type strain, but it was reused by the cell after 4 h of cultivation. 1.2 and 1.9 g/L of acetate were produced by *K. pneumoniae* ∆*ptsG* and *K. pneumoniae* ∆*crr*, and no reuse occurred during the experimental period. The final concentration of 2,3-BDO was 5 g/L in experiments performed with all these strains.

### Blocking the glycerol oxidation branch of *Dha* pathway

The yields of 1,3-PDO from glycerol were 0.36 and 0.48 mol/mol for *K. pneumoniae* ∆*ptsG* and *K. pneumoniae* ∆*crr*. It indicated that, like the wild-type strain, part of the glycerol was oxidised by the cell. Here, the glycerol oxidation branch of *dha* pathway was blocked to reduce glycerol oxidation.

**Fig. 3:**
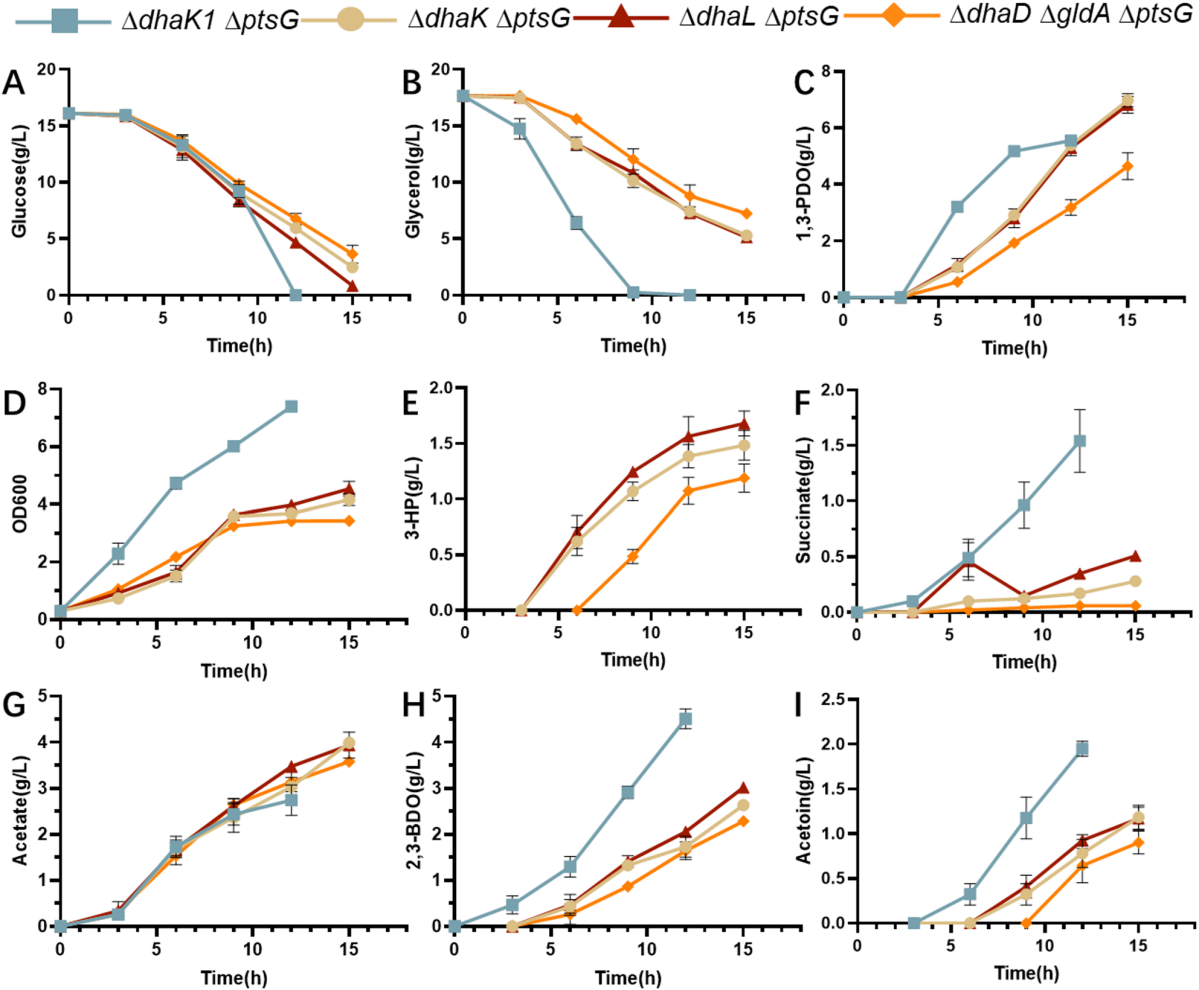
Cell growth, substrates (glucose and glycerol) depletion, product (1,3-PDO) and by-products (lactate, succinate, acetate, 2,3-BDO and acetoin) formation during the glycerol oxidation pathway blocked strains. *K. pneumoniae* ∆*dhaK1*∆*ptsG*, *K. pneumoniae* ∆*dhaK*∆*ptsG*, *K. pneumoniae* ∆*dhaL*∆*ptsG* and *K. pneumoniae* ∆*dhaD*∆*gldA*∆*ptsG*. Values are presented as the mean ± standard error (*n* = 3)

The first reaction of the oxidation pathway was catalysed by glycerol dehydrogenase. *dhaD* and *gldA* encode isoenzymes of glycerol dehydrogenase. *dhaKLM* encodes the dihydroxyacetone kinase Ⅱ, which is responsible for the second step of glycerol oxidation. *dhaK1* encoded the dihydroxyacetone kinase I. These genes were knocked out individually to block the oxidation branch of *dha* pathway based on the CCR-deficient strain. *K. pneumoniae* ∆*dhaK1*∆*ptsG K. pneumoniae* ∆*dhaK*∆*ptsG*, *K. pneumoniae* ∆*dhaL*∆*ptsG*, and *K. pneumoniae* ∆*dhaD*∆*gldA*∆*ptsG* were cultivated in shake flasks with glucose and glycerol as co-substrates. The results are presented in Fig. 3.

The performance of *K. pneumoniae* ∆*dhaK1*∆*ptsG* was close to that of *K. pneumoniae* ∆*ptsG* (shown in Fig. 2). 17.3 g/L of glycerol was consumed after 9 h of cultivation, and 5.6 g/L of 1,3-PDO was produced. Glucose was exhausted after 12 h of cultivation. The final cell density was 7.4 OD units. The substrate consumption rates, 1,3-PDO and by-products of *K. pneumoniae* ∆*dhaK1*∆*ptsG* were all close to that of *K. pneumoniae* ∆*ptsG*. Thus, knocking out of *dhaK1* does not affect the glycerol oxidation.

*K. pneumoniae* ∆*dhaK*∆*ptsG*, *K. pneumoniae* ∆*dhaL*∆*ptsG*, and *K. pneumoniae* ∆*dhaD*∆*gldA*∆*ptsG* exhibited similar performances. Glucose was exhausted after 15 h of cultivation of *K. pneumoniae* ∆*dhaL*∆*ptsG*. While at this time, 2.5 and 3.7 g/L of glucose remained in the broth of *K. pneumoniae* ∆*dhaK*∆*ptsG* and *K. pneumoniae* ∆*dhaD*∆*gldA*∆*ptsG*. The glucose consumption rates of these strains were all slower than that of *K. pneumoniae* ∆*ptsG* (shown in Fig. 2). Glycerol consumption by these strains proceeded very slowly. 12.4, 12.5 and 10.4 g/L of glycerol were consumed by *K. pneumoniae* ∆*dhaK*∆*ptsG*, *K. pneumoniae* ∆*dhaL*∆*ptsG* and *K. pneumoniae* ∆*dhaD*∆*gldA*∆*ptsG* after 15 h of cultivation. 6.9, 6.8 and 4.6 g/L of 1,3-PDO were produced by these strains. The yields of 1,3-PDO from glycerol were 0.67, 0.66 and 0.54 mol/mol, respectively. These results indicated that the glycerol oxidation pathway was blocked in these strains, and the yields of 1,3-PDO from glycerol were improved. However, the 1,3-PDO productivities changed very slowly. This indicated that the 1.3-PDO synthesis pathway of these strains was in low activity.

The final cell densities of these strains were 4.2, 4.5 and 3.4 OD units. These values were lower than those of *K. pneumoniae* ∆*ptsG* (shown in Fig. 2). These results indicated that cell growth was inhibited by blocking the pathway of glycerol oxidation. Succinate, 2,3-BDO and acetoin produced by these three strains were all lower than that of *K. pneumoniae* ∆*ptsG* or *K. pneumoniae* ∆*dhaK1*∆*ptsG*. Acetate levels of these strains were increased. A remarkable difference of these three strains to *K. pneumoniae* ∆*ptsG* was that 3-hydroxypropionic acid (3-HP) was produced, with the titers of 1.5, 1.7 and 1.2 g/L. 3-HP is an oxidation product of 3-HPA.

### Blocking the glycerol oxidation pathway by knocking out *DhaM*

**Fig. 4:**
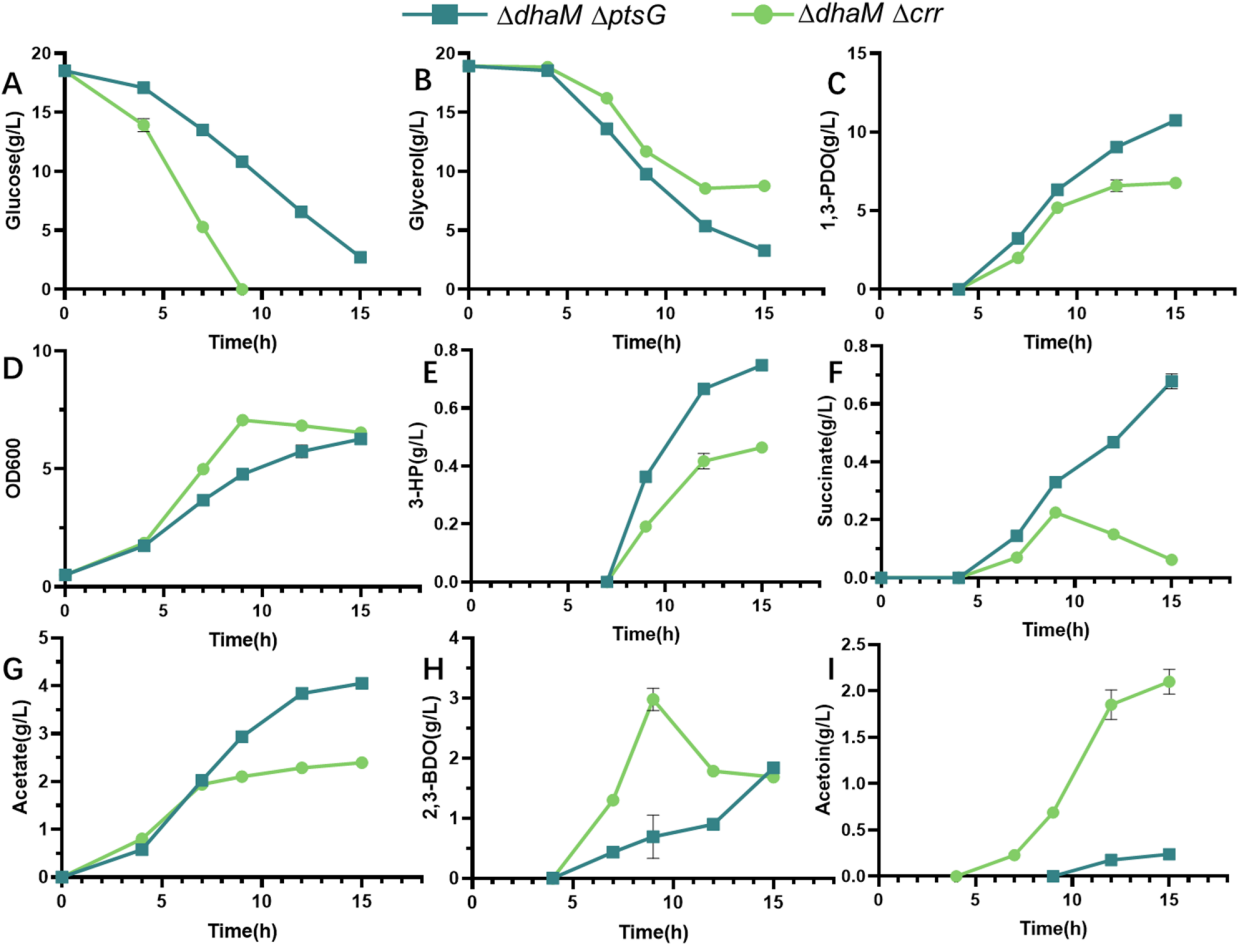
Cell growth, substrates (glucose and glycerol) depletion, product (1,3-PDO) and by-products (lactate, succinate, acetate, 2,3-BDO and acetoin) formation during *dhaM* knocked-out strains. *K. pneumoniae* ∆*dhaM*∆*ptsG* and *K. pneumoniae* ∆*dhaM*∆*crr*. Values are presented as the mean ± standard error (*n* = 3)

*dhaM* encodes the large subunit of dihydroxyacetone kinase Ⅱ. The *dhaM* knocked-out strain had little effect on the activities of key enzymes for 1,3-PDO synthesis in our previous research [18]. Here, *K. pneumoniae* ∆*dhaM*∆*ptsG* and *K. pneumoniae* ∆*dhaM*∆*crr* were constructed and cultivated in shake flasks with glucose and glycerol as co-substrates. The results are presented in Fig. 4.

Glucose was depleted after 9 h of cultivation of *K. pneumoniae* ∆*dhaM*∆*crr.* 7.2 g/L of glycerol was consumed during this period, and 5.2 g/L of 1,3-PDO was produced. After that, cell growth, glycerol consumption and 1,3-PDO production all very slow. The final titer of 1,3-PDO was 6.8 g/L, with a yield of 0.80 mol/mol glycerol. By-products of the process were 3-HP (0.5 g/L), succinate (0.06 g/L), 2,3-BDO (1.7 g/L) and acetoin (2.1 g/L). This demonstrates that when *K. pneumoniae* ∆*dhaM*∆*crr* is cultivated with dual carbon sources, glucose plays a significant role in cell growth and 1,3-PDO synthesis from glycerol.

The glucose consumption rate of *K. pneumoniae* ∆*dhaM*∆*ptsG* was slower than that of *K. pneumoniae* ∆*dhaM*∆*crr*. Whereas its glycerol consumption rate was fast. 15.8 g/L of glucose and 15.6 g/L of glycerol were consumed after 15 h of cultivation. 10.7 g/L of 1,3-PDO was produced, with a yield of 0.83 mol/mol glycerol. Compared to *K. pneumoniae* ∆*dhaM*∆*crr*, the production of acetoin and 2,3-BDO was decreased. Other by-products, including 3-HP, succinate, and acetate, were all increased, and with the levels of 0.7 g/L, 0.7 g/L, and 4.1 g/L, respectively.

The fermentation data of constructed strains were summarised in Table 1. It can be determined that *K. pneumoniae* ∆*dhaM*∆*ptsG* achieved a high yield and high final 1,3-PDO titer, and was therefore selected for further study.

**Table 1:** 1,3-PDO production by *K. pneumoniae* strains using glucose and glycerol as co-substrates

Strains	Time (h)	Glycerol consumed (g/L)	1,3-PDO levels (g/L)	Yield (mol/mol glycerol)
Wild-type	12	19.2	6.0	0.37
∆*crr*	12	18.4	7.3	0.48
∆*ptsG*	12	19.3	5.8	0.36
∆*dhaK1*∆*ptsG*	9	17.3	5.6	0.38
∆*dhaK*∆*ptsG*	15	12.4	6.9	0.67
∆*dhaL*∆*ptsG*	15	12.5	6.8	0.66
∆*dhaD*∆*gldA*∆*ptsG*	15	10.4	4.6	0.54
∆*dhaM*∆*crr*	15	10.2	6.8	0.80
∆*dhaM*∆*ptsG*	15	15.6	10.7	0.83

### Further block the glycerol oxidation pathways

**Fig. 5:**
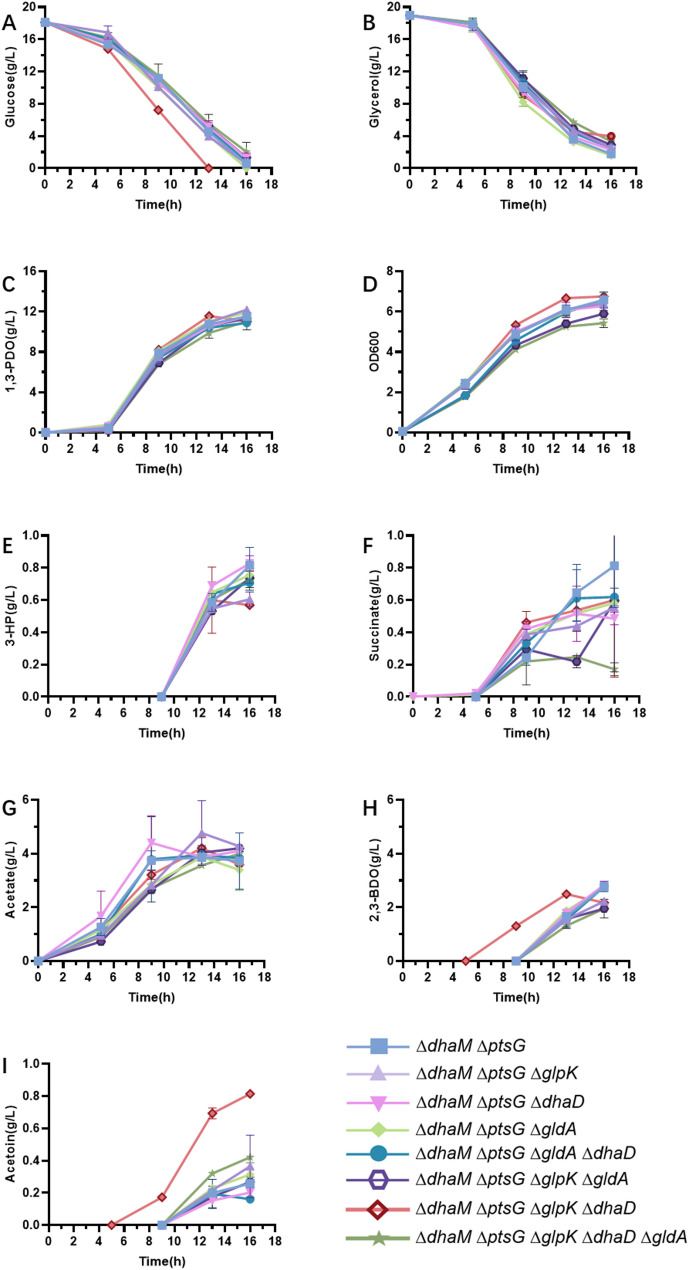
Cell growth, substrates (glucose and glycerol) depletion, product (1,3-PDO) and by-products (lactate, succinate, acetate, 2,3-BDO and acetoin) formation during glycerol oxidation pathways blocked strains. *K. pneumoniae* ∆*dhaM*∆*ptsG*, *K. pneumoniae* ∆*dhaM*∆*ptsG*∆*glpK*, *K. pneumoniae* ∆*dhaM*∆*ptsG*∆*dhaD*, *K. pneumoniae* ∆*dhaM*∆*ptsG*∆*gldA*, *K. pneumoniae* ∆*dhaM*∆*ptsG*∆*gldA*∆*dhaD*, *K. pneumoniae* ∆*dhaM*∆*ptsG*∆*glpK*∆*gldA*, *K. pneumoniae* ∆*dhaM*∆*ptsG*∆*glpK*∆*dhaD* and *K. pneumoniae* ∆*dhaM*∆*ptsG*∆*glpK*∆*dhaD*∆*gldA*. Values are presented as the mean ± standard error (*n* = 3)

*K. pneumoniae* ∆*dhaM*∆*ptsG* had a 1,3-PDO yield of 0.83 mol/mol glycerol, which remained lower than the theoretical maximum value. Thus, some glycerol remains oxidised by the cell. To further improve the yield, glycerol oxidation through other pathways needs to be blocked. *glpK* encodes the glycerol kinase, which catalyses the first step of the glycerol oxidation in the *glp* pathways. *gldA* and *dhaD* encode isoenzymes of glycerol dehydrogenases, which are located in the first step of the *dha* pathway. These genes were knocked out individually and combined based on *K. pneumoniae* ∆*dhaM*∆*ptsG*. These constructed strains were cultured using glucose and glycerol as co-substrates in shake flasks, and the results are presented in Fig. 5.

**Fig. 6:**
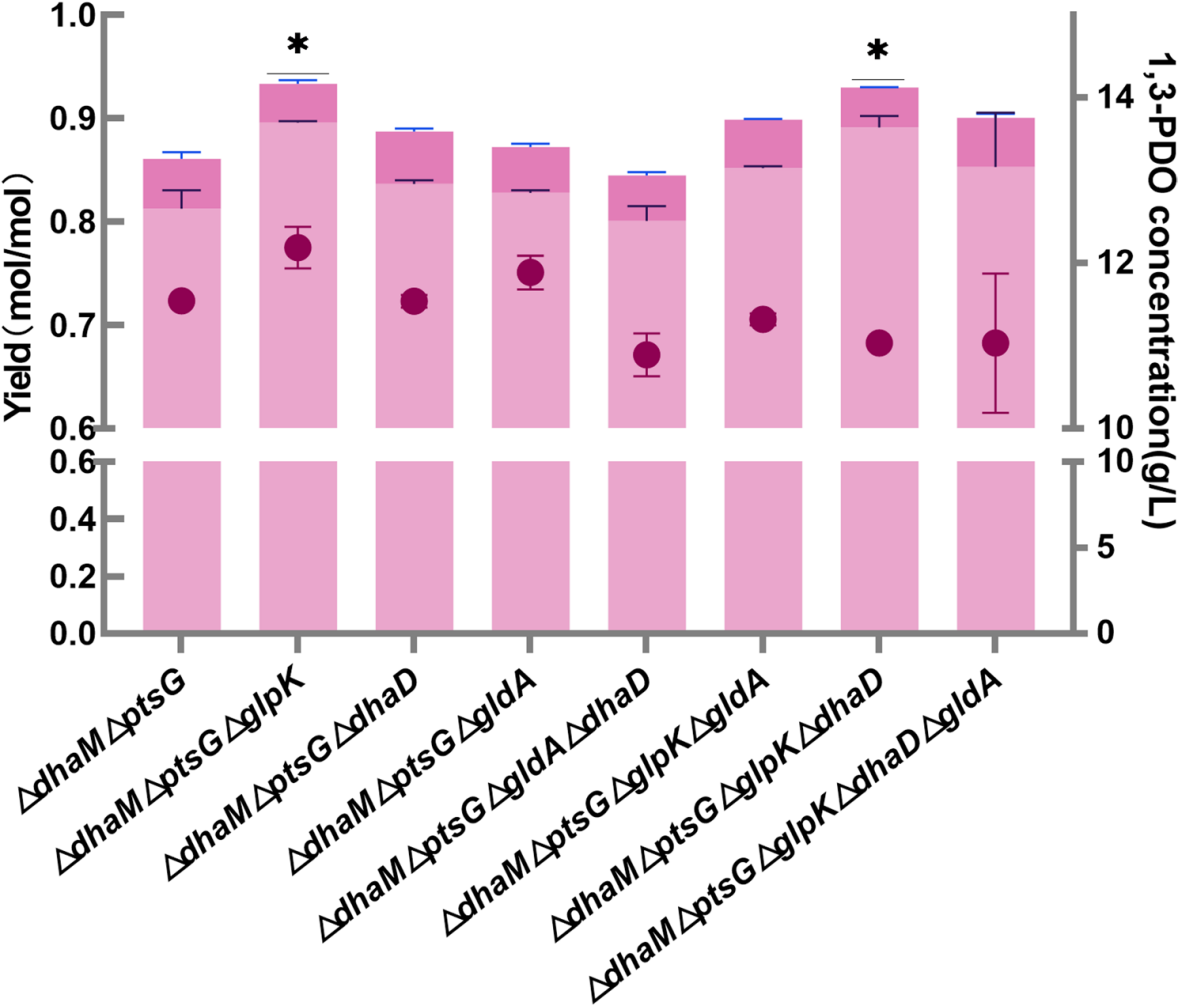
1,3-PDO and 3-HP formation of strains that inactivated enzymes related to glycerol oxidation pathways. Values are presented as the mean ± standard error (*n* = 3)

Cell growth, glucose consumption, glycerol consumption, and 1,3-PDO production showed similar trends among these strains. It indicated that knocking out *glpK*, *dhaD*, or *gldA* had little impact on the growth of the cell under the co-substrate process. The final levels of 1,3-PDO and 3-HP varied among these strains, and they were summarised in Fig. 6.

*K. pneumoniae* ∆*dhaM*∆*ptsG* consumed 14.8 g/L of glucose and 16.1 g/L of glycerol after 16 h of cultivation, 11.5 g/L of 1,3-PDO and 0.80 g/L of 3-HP were produced. The yields of glycerol to 1,3-PDO and 3-HP were 0.80 and 0.05 mol/mol, respectively. The combined yield of glycerol to the two chemicals was 0.85 mol/mol. The yields of 1,3-PDO and the sum yields of 1,3-PDO and 3-HP of these strains all improved compared with *K. pneumoniae* ∆*dhaM*∆*ptsG* except for *K. pneumoniae* ∆*dhaM*∆*ptsG*∆*gldA*∆*dhaD*. Compared *glpK* wild type and knocked out strains, *K. pneumoniae* ∆*dhaM*∆*ptsG*∆*glpK* (0.90 mol/mol glycerol) to *K. pneumoniae* ∆*dhaM*∆*ptsG* (0.82 mol/mol glycerol), *K. pneumoniae* ∆*dhaM*∆*ptsG*∆*glpK*∆*gldA* (0.89 mol/mol glycerol) to *K. pneumoniae* ∆*dhaM*∆*ptsG*∆*gldA* (0.83 mol/mol glycerol), *K. pneumoniae* ∆*dhaM*∆*ptsG*∆*glpK*∆*dhaD* (0.89 mol/mol glycerol) to *K. pneumoniae* ∆*dhaM*∆*ptsG*∆*dhaD* (0.84 mol/mol glycerol), and *K. pneumoniae* ∆*dhaM*∆*ptsG*∆*glpK*∆*dhaD*∆*gldA* (0.86 mol/mol glycerol) to *K. pneumoniae* ∆*dhaM*∆*ptsG*∆*dhaD*∆*gldA* (0.85 mol/mol glycerol), the 1,3-PDO yields were increased in all cases.

Compared *gldA* or *dhaD* wild type and knocked out strains, *K. pneumoniae* ∆*dhaM*∆*ptsG*∆*gldA* (0.83 mol/mol glycerol, 11.89 g/L) and *K. pneumoniae* ∆*dhaM*∆*ptsG*∆*dhaD* (0.83 mol/mol glycerol, 11.55 g/L) to *K. pneumoniae* ∆*dhaM*∆*ptsG* (0.82 mol/mol glycerol, 11.54 g/L), the 1,3-PDO yields were increased in both cases. These results indicated that GldA and DhaD both contributed to the conversion of glycerol to dihydroxyacetone. However, when the two enzymes were inactive together, the yields and the final titer of 1,3-PDO were reduced in *K. pneumoniae* ∆*dhaM*∆*ptsG*∆*dhaD*∆*gldA* (0.80 mol/mol, 10.89 g/L). Suggesting that completely blocking the glycerol dehydrogenase pathway was detrimental to the synthesis of 1,3-PDO.

Among all these strains, *K. pneumoniae* ∆*dhaM*∆*ptsG*∆*glpK* achieved the highest yield and final 1,3-PDO titer. This strain was used for further investigations.

### Optimisation of the ratio of glucose to glycerol in the medium

**Fig. 7:**
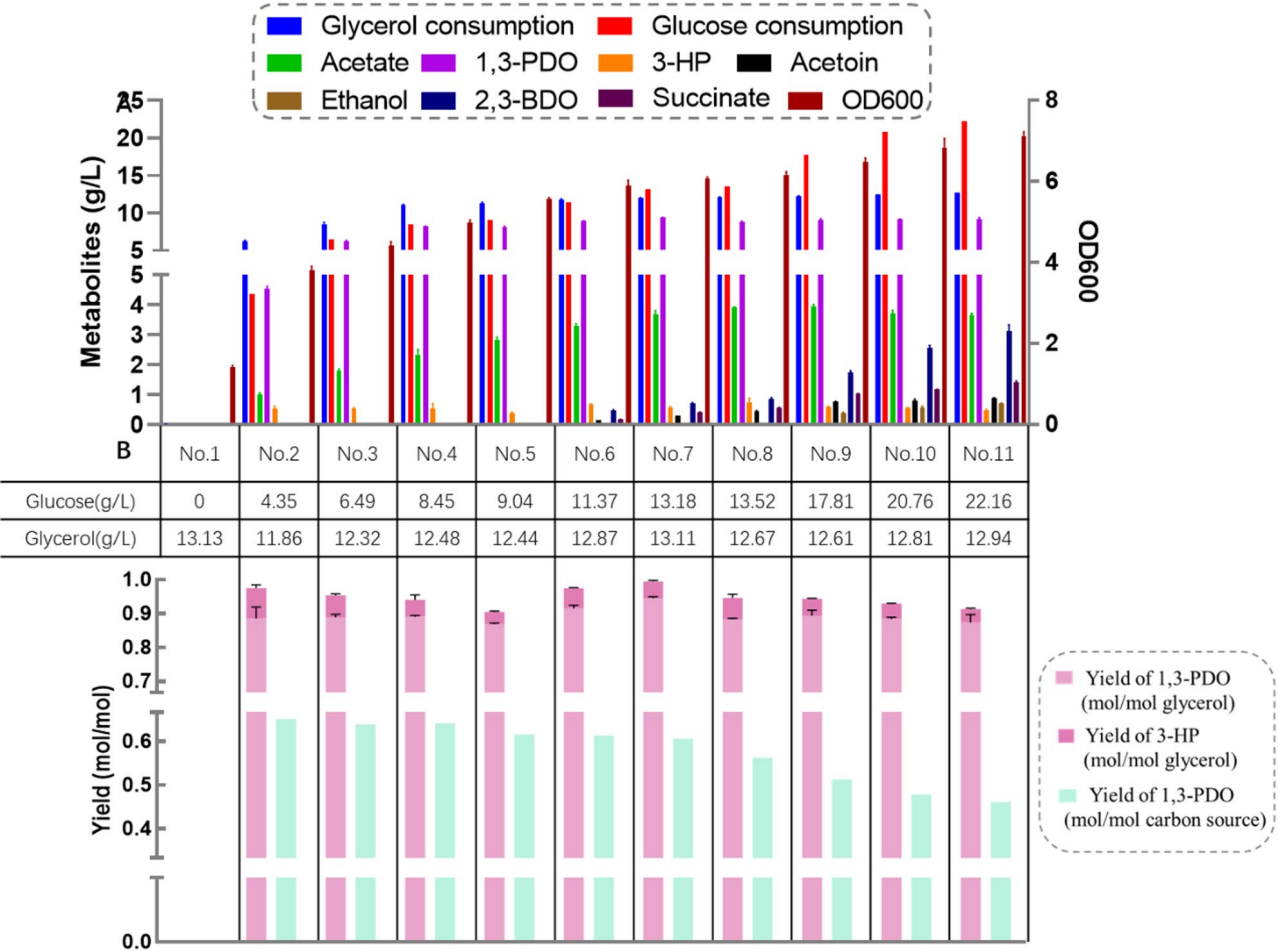
(A) Cell growth, glycerol depletion, product (1,3-PDO), by-products (3-HP, 2,3-BDO, acetoin, succinate, acetate, ethanol) formation and (B) Yields of glycerol to 1,3-PDO and 3-HP of *K. pneumoniae* ∆*dhaM*∆*ptsG*∆*glpK* with various glucose concentrations in the media. Values are presented as the mean ± standard error (*n* = 3)

The growth and metabolism of *K. pneumoniae* ∆*dhaM*∆*ptsG*∆*glpK* require both glucose and glycerol as carbon sources. For the purpose of avoiding the energy and NADH waste caused by an excess of glucose, the ratio of glucose to glycerol in the medium was optimised. Glycerol in these media was 13 g/L, and glucose concentrations were various, as listed in Table 2. Fermentation results are shown in Fig. 7.

**Table 2:** Glucose and glycerol concentrations in the media for cultivation of *K. pneumoniae* ∆*dhaM*∆*ptsG*∆*glpK*

Media	No.1	No.2	No.3	No.4	No.5	No.6	No.7	No.8	No.9	No.10	No.11
Glucose (g/L)	0	4.35	6.49	8.45	9.04	11.37	13.18	13.52	17.81	20.76	22.16
Glycerol (g/L)	13.13	11.86	12.32	12.48	12.44	12.87	13.11	12.67	12.61	12.81	12.94

With the increase in glucose concentration in the media, cell densities increased. The highest cell density was 7.1 OD units with a glucose concentration of 22.2 g/L. Glycerol consumption and 1,3-PDO titers both increased with the glucose concentrations in the range of 0–11 g/L. Glycerol consumption and 1,3-PDO titer were no longer increased after glucose concentrations above 11 g/L. The highest 1,3-PDO titer was 9.4 g/L, achieved with 13 g/L of glucose in the medium. 3-HP and acetate had similar tendencies with 1,3-PDO. Their levels increased in the range of 0–11 g/L of glucose and remained stable after glucose levels were higher than 11 g/L. Other by-products, including 2,3-BDO, acetoin, succinate, and ethanol produced in the broth, all showed a positive relationship with the glucose concentration in the test ranges.

Notably, cells could not grow in medium No. 1 (no glucose), confirming that *K. pneumoniae* ∆*dhaM*∆*ptsG*∆*glpK* cannot utilise glycerol as the sole carbon source for growth. The ratio of glucose to glycerol concentration was below 2:3 in media No. 2–4, and glycerol in these media was not completely consumed. Few by-products were formed, except low levels of acetic acid and 3-HP. These media had high carbon source conversion rates, which were 0.65, 0.64 and 0.64 mol/mol carbon source (glycerol plus glucose), respectively. It indicates that NADH and energy provided by glucose catabolism were almost entirely used for 1,3-PDO synthesis and cell growth. After the ratio of glucose to glycerol in the medium exceeded 2:3, NADH and energy generated by glucose metabolism were excessive, leading to an increase in by-products. The yields of glycerol to 1,3-PDO and 3-HP in different medium glucose concentrations are shown in Fig. 7B.

The No. 7 medium contains the same concentration of glucose and glycerol (13 g/L), and provides the highest yield of glycerol to 1,3-PDO. The yields of glycerol to 3-HP and 1,3-PDO were 0.05 and 0.95 mol/mol glycerol, respectively. The sum yield of the two chemicals was 1.00 mol/mol glycerol and 0.61 mol/mol carbon source. This indicates that after the inactivation of glycerol kinase and dihydroxyacetone kinase II, the oxidative pathway of glycerol was completely blocked, and glycerol was directed entirely towards the reductive pathway for 1,3-PDO synthesis. 1,3-PDO and 3-HP were the metabolites of the glycerol reductive pathway. Therefore, the ratio of glucose to glycerol concentration below 2:3 was the most energy-efficient way to synthesise 1,3-PDO, and the ratio of glucose to glycerol concentration 1:1 in weight was the optimal value for 1,3-PDO production.

The main by-product of the No.2- No.7 medium was acetate. Stoichiometrically, 2 molecules of acetate are formed from 1 molecule of glucose, with 2 NADH generated net in the process. 1 molecule of 1,3-PDO was formed from glycerol and consumed 1 NADH. Thus, the ratio of glucose to glycerol at 0.5:1 mol/mol (1:1 in weight) leads to a balance of NADH generation and consumption. This coincides with the optimal value obtained in the flask experiments. A lower ratio of glucose to glycerol requires more NADH generation to keep the balance. This needs glucose catabolism through the TCA cycle. Exceeding glucose resulted in high levels of 2,3-BDO, acetoin, succinate, and ethanol formation.

### Optimisation of air supply in bioreactors

**Fig. 8:**
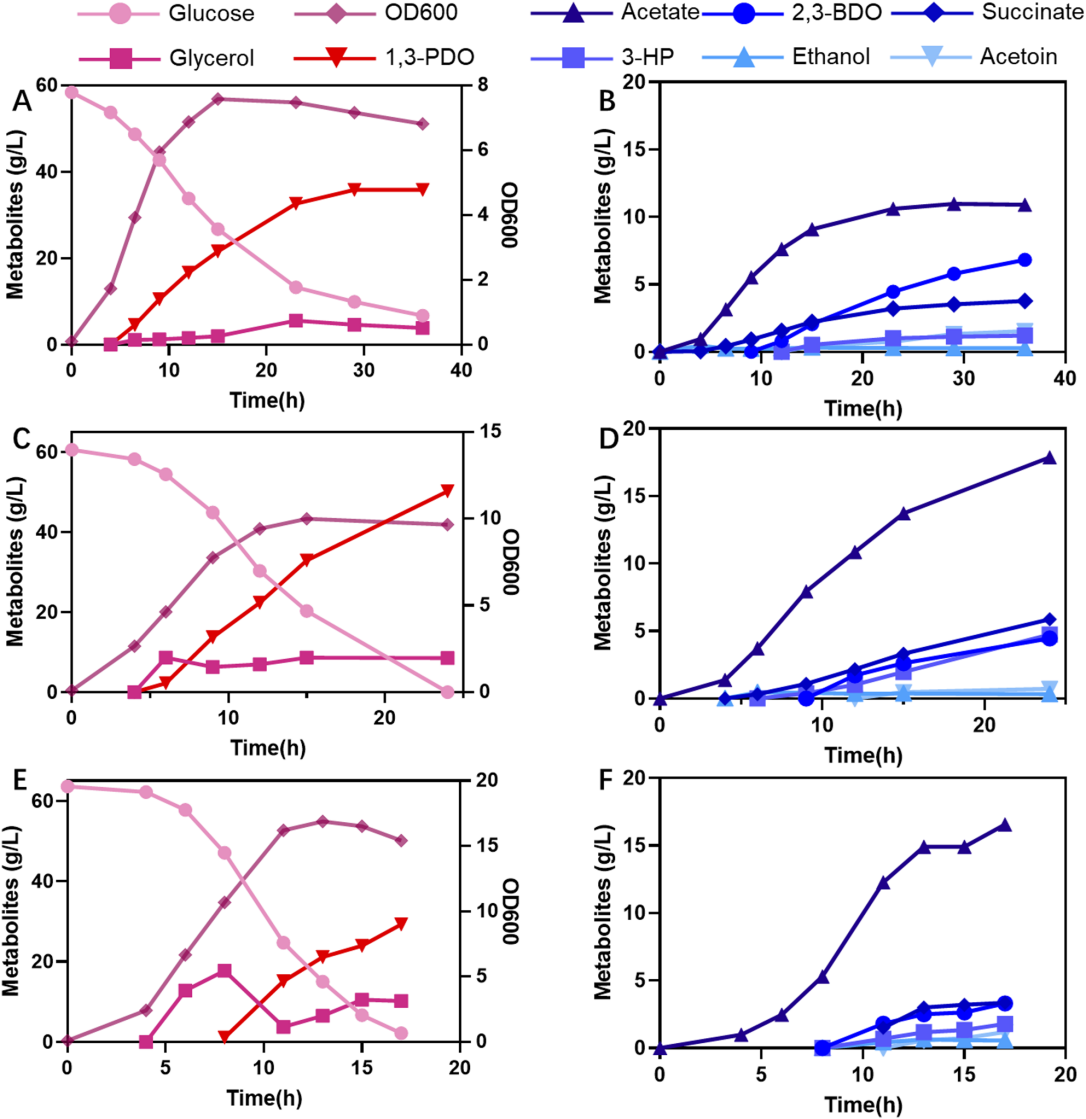
Cultivation of *K. pneumoniae* ∆*dhaM*∆*ptsG*∆*glpK* in bioreactors at various air supply conditions; (A) (B), 0 L/min, 200 rpm, (C) (D), 2 L/min, 200 rpm, (E) (F), 4 L/min, 400 rpm. (Process conditions: T = 37 °C, pH = 7.0, V = 3 L)

Air supply is an important parameter that affects the physiological characteristics of cells. Batch fermentations with different air supply were done in 5 L bioreactors for the cultivation of *K. pneumoniae* ∆*dhaM*∆*ptsG*∆*glpK* using glucose and glycerol as co-substrates. One process was performed without the air supply to give an anaerobic condition, while the others were performed at microaerobic and aerobic conditions with airflow rates of 2 L/min and 4 L/min, respectively. The results are shown in Fig. 8.

As shown in Fig. 8A and B, after 36 h of cultivation, 51.61 g/L of glucose and 47.14 g/L of glycerol were consumed, yielding 35.83 g/L of 1,3-PDO. The 1,3-PDO yield and productivity were 0.92 mol/mol and 1.00 g/L/h, respectively. The highest cell density was 7.6 OD units. By-products were 10.90 g/L of acetate, 3.77 g/L of succinate, 6.83 g/L of 2,3-BDO, 1.20 g/L of 3-HP, 0.27 g/L of ethanol and 1.54 g/L of acetoin.

Figure 8C and D showed that the process operated in a low air supply condition. Glucose and glycerol consumption rates were both higher than those in no airflow conditions (Fig. 8A, B). 60.50 g/L of glucose and 64.81 g/L of glycerol were consumed, and 50.25 g/L of 1,3-PDO was produced after 24 h of cultivation. Compared to the process under no airflow conditions, the conversion of glycerol to 1,3-PDO and cell density were both increased, with values of 0.938 mol/mol and 10.0 OD units. The 1,3-PDO productivity was 2.09 g/L/h, twice that of the no airflow conditions. 17.88 g/L of acetate, 5.88 g/L of succinate, 4.45 g/L of 2,3-BDO, 4.73 g/L of 3-HP and 0.73 g/L of acetoin were produced. Apart from 2,3-BDO and acetoin, the concentrations of other metabolites were all higher than those produced in the no airflow conditions.

The air supply was further increased in the process shown in Fig. 8E and F. 61.42 g/L of glucose and 39.12 g/L of glycerol were consumed, and 29.26 g/L of 1,3-PDO was produced after 17 h of cultivation. The highest cell density was 16.9 OD units. Acetate remained the main by-product of the process, with a titer of 16.55 g/L. 3.35 g/L of succinate, 3.30 g/L of 2,3-BDO, 1.79 g/L of 3-HP and 1.19 g/L of acetoin were produced. The yields of glycerol to 1,3-PDO of the three processes are shown in Table 3.

**Table 3:** 1,3-PDO production in batch processes with various air supply

air supply	Time (h)	Substrate consumption		1,3-PDO			
		Glucose (g/L)	Glycerol (g/L)	Titer (g/L)	Yield (mol/mol glycerol)	Yield (mol/mol carbon source)	Productivity (g/L/h)
Anaerobic	36	51.61	47.14	35.83	0.920	0.590	1.00
Microaerobic	24	60.50	64.81	50.25	0.938	0.635	2.09
Aerobic	17	61.42	39.12	29.26	0.905	0.502	1.72

As shown in Table 3, glucose consumption rates increased with the air supply. However, the high air supply condition was unfavourable for 1,3-PDO synthesis. 64.81 g/L of glycerol was consumed in microaerobic conditions, while 39.12 g/L of glycerol was consumed in aerobic conditions. The yield of 1,3-PDO was also affected by the air supply, and the microaerobic condition was favourable for 1,3-PDO synthesis. The yield of glycerol to 1,3-PDO (0.938 mol/mol) closely approached the theoretical maximum. Although the glucose consumption rate and cell growth were fast under higher air supply conditions, the synthesis of 1,3-PDO was inhibited. It indicates that microaerobic conditions are favourable for 1,3-PDO production than anaerobic or aerobic conditions.

### Fed-batch fermentation

Fed-batch fermentations were performed in 5 L bioreactors with an airflow rate of 2 L/min and agitation of 200 rpm. During the process, 3-hydroxypropionaldehyde (3-HPA), which is an intermediate in 1,3-PDO synthesis from glycerol and a toxic chemical, accumulated in the broth. A high level of glycerol in the culture medium leads to the accumulation of 3-HPA. Therefore, glycerol in the fed-batch cultivation was controlled at 10 g/L, while the glucose was fed at 58 h of cultivation.

**Fig. 9:**
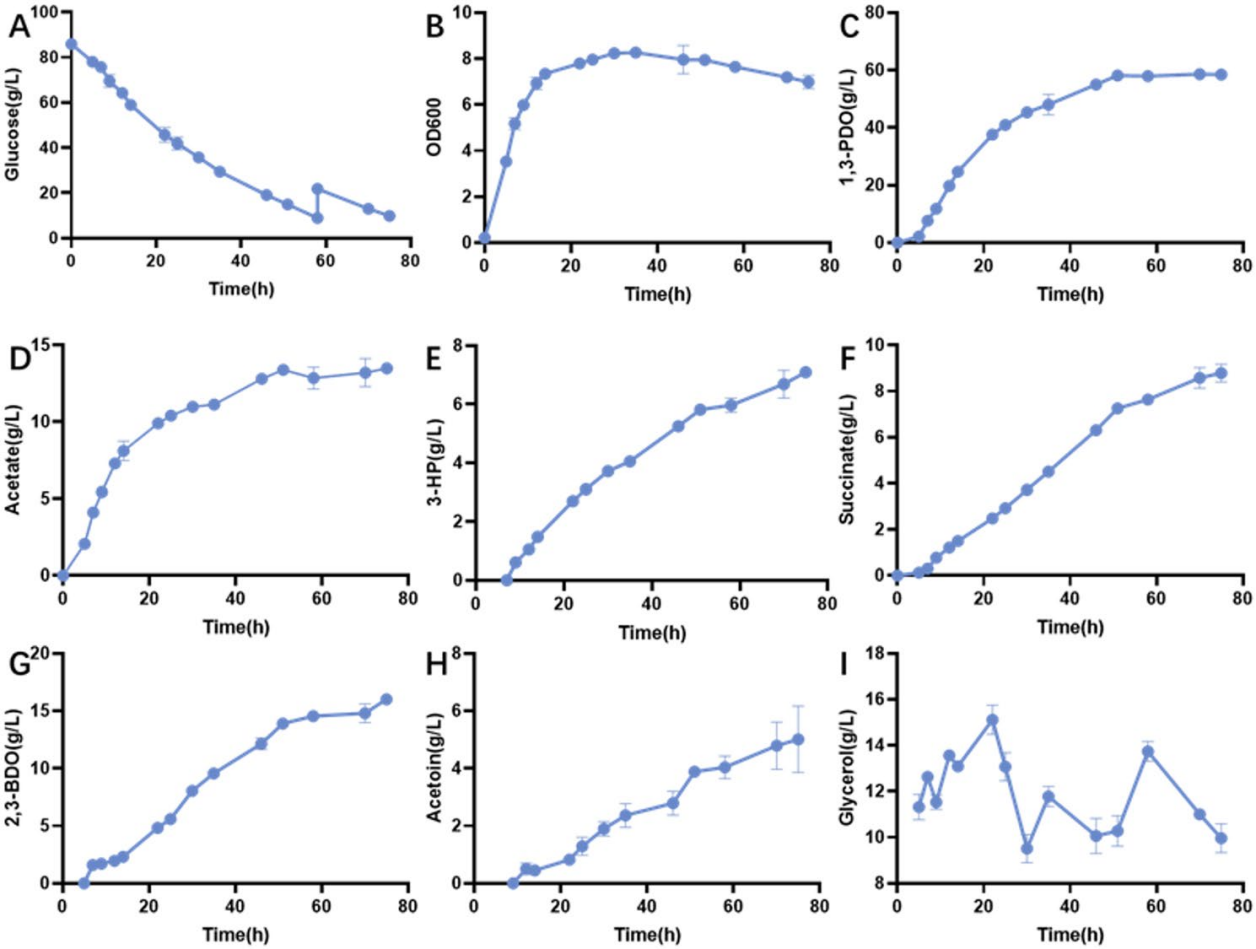
Fed-batch experiments of *K. pneumoniae* ∆*dhaM*∆*ptsG*∆*glpK* in a bioreactor: Cell growth, glucose consumption, product (1,3-PDO) and byproducts (Acetate, 3-HP, succinate, 2,3-BDO and acetoin) formation (Process conditions: *T* = 37 °C; pH = 7.0, V _initial_ = 3 L; Airflow: 2 L/min, *n* = 200 rpm; Values are presented as the mean ± standard error (*n* = 3)

Glucose consumption rates were stable at 1.4 g/L/h. Glucose was fed into the broth after 58 h of cultivation, when the glucose in the medium dropped to 8.9 g/L. As shown in Fig. 9B, cells were rapidly growing in the 14 h of cultivation, and the cell density reached 7.3 OD units. Cell growth entered the stationary phase after 30 h, and the cell density reached 8.3 OD units. 1,3-PDO in the broth increased continuously during the first 50 h. However, in the later stages of cultivation, the 1,3-PDO synthesis was stopped. 58.7 g/L of 1,3-PDO was produced after 70 h of cultivation (Fig. 9C). The yield of 1,3-PDO was 0.93 mol/mol glycerol and 0.63 mol/mol carbon source, with a productivity of 0.84 g/L/h. The tendency of acetate production was similar to that of 1,3-PDO. Acetate entered a stable phase after 50 h of cultivation, with a final level of 13 g/L. Other by-products, including 3-HP, succinate, 2,3-BDO, and acetoin, were produced in the broth and increased continuously during the process. Their final levels were 7.1, 8.8, 16.0 and 5.0 g/L, respectively.

## Discussion

### Knocking out of *DhaM* was the sole feasible solution to block the oxidation branch of the *Dha* pathway, while maintaining the activity of the *Dha* Operon

1,3-PDO is synthesised through the *dha* pathway. In the oxidised branch of *dha* pathway, glycerol dehydrogenase isoenzymes (*gldA*, *dhaD*) catalyse the conversion of glycerol to dihydroxyacetone. Dihydroxyacetone is phosphorylated and flows into glycolysis for further catabolism. The main purpose of this research is to block the glycerol oxidation pathways and direct all glycerol for 1,3-PDO synthesis. The oxidation branch of *dha* pathway was blocked by knocking out key genes of the operon. The reaction of glycerol to dihydroxyacetone was blocked in *K. pneumoniae* ∆*dhaD*∆*gldA*∆*ptsG.* The reaction of dihydroxyacetone phosphate formation from glycerol was blocked in *K. pneumoniae* ∆*dhaK*∆*ptsG* and *K. pneumoniae* ∆*dhaL*∆*ptsG*. However, 1,3-PDO produced by these strains with glycerol and glucose as co-substrates was all at low levels (Fig. 3).

The underlying mechanism involves impaired expression of the *dha* operon. The expression of the *dha* operon is induced by dihydroxyacetone (DHA), and thus named [28]. Dihydroxyacetone cannot be synthesised in *K. pneumoniae* ∆*dhaD*∆*gldA*∆*ptsG.* Thus, the expression of the *dha* operon was not induced, resulting in low 1,3-PDO levels.

Besides catalysing dihydroxyacetone phosphate formation, dihydroxyacetone kinase II was a regulatory protein of the *dha* operon. Dihydroxyacetone kinase II interacts with a regulatory protein DhaR, and activates the expression of this operon when dihydroxyacetone is present. In the reaction of dihydroxyacetone phosphate formation from dihydroxyacetone, the phosphate group from PEP is sequentially transferred through *DhaM*, DhaL, DhaK and finally to dihydroxyacetone. The unphosphorylated form of DhaL contacts with DhaR to activate the *dha* operon expression [29]. DhaL was lost in *K. pneumoniae* ∆*dhaL*∆*ptsG*, so the *dha* operon expression was inhibited. DhaL is maintained in a phosphorylated form in *K. pneumoniae* ∆*dhaK*∆*ptsG*, due to the absence of DhaK. Thus, the *dha* operon could not be induced. The *dha* operon expression was affected in the two strains and resulted in low 1,3-PDO levels (Fig. 3).

In *K. pneumoniae* ∆*dhaM*, where DhaM is absent, no phosphate group is transfered to DhaL, leaving it in an unphosphorylated state. Thus, the *dha* operon can be induced by dihydroxyacetone in *K. pneumoniae* ∆*dhaM.* In our previous research about dihydroxyacetone kinases, it was found that key enzymes of the *dha* pathway were kept active in *K. pneumoniae* ∆*dhaM* [18]. In this research, *K. pneumoniae* ∆*dhaMA*∆*ptsG* exhibited high 1,3-PDO titers when cultured with glycerol and glucose as co-substrates (Fig. 4). This agrees with our previous research about *dhaM* knocked out strains [18].

Besides dihydroxyacetone kinases II, there is an ATP-dependent dihydroxyacetone kinase I in the *dha* operon, and it is encoded by *dhaK1*. This enzyme showed no contribution to dihydroxyacetone phosphate formation in our previous research [18]. In this research, *K. pneumoniae* ∆*dhaK1*∆*ptsG* showed no difference to *K. pneumoniae* ∆*ptsG* in 1,3-PDO production with glycerol and glucose as co-substrates (Fig. 3). This further confirms that ATP-dependent dihydroxyacetone kinase I is inactive in *K. pneumoniae*.

### Knocking out of *GlpK* increased the yield of 1,3-PDO from glycerol

The *dha* pathway is the main route of glycerol oxidation pathways. When the glycerol dehydrogenase isoenzymes (*gldA*, *dhaD*) were inactive, the obtained strains were unable to grow in a minimal medium with glycerol as the sole carbon source [7]. In this research, *K. pneumoniae* ∆*dhaD*∆*gldA*∆*ptsG* was constructed. Compared with *K. pneumoniae* ∆*ptsG*, both cell density and the substrate consumption rates of *K. pneumoniae* ∆*dhaD*∆*gldA*∆*ptsG* were significantly reduced when cultured using glucose and glycerol as co-substrates (Figs. 5 and 6).

Besides the *dha* pathway, there is a *glp* pathway that oxidises glycerol. The *dha* pathway is a fermentative route, while the *glp* pathway is a respiratory route. Glycerol oxidation via the respiration route requires oxygen or nitrate as an electron acceptor [19]. Inactivating glycerol kinase (GlpK) does not significantly affect the growth of *Escherichia coli* with glycerol as the sole carbon source [30]. Thus, *glp* pathway seems not as important as *dha* pathway for glycerol oxidation.

In this research, the *glp* pathway was blocked by knocking out *glpK*. Engineered strains, including *K. pneumoniae* ∆*dhaM*∆*ptsG*∆*glpK*, *K. pneumoniae* ∆*dhaM*∆*ptsG*∆*glpK*∆*gldA*, *K. pneumoniae* ∆*dhaM*∆*ptsG*∆*glpK*∆*dhaD*, and *K. pneumoniae* ∆*dhaM*∆*ptsG*∆*glpK*∆*dhaD*∆*gldA*, all showed an increase in the 1,3-PDO yields. This suggests that some glycerol is indeed oxidised through the *glp* pathway. The sum yield of glycerol to 1,3-PDO and 3-HP was 0.94 mol/mol glycerol in *K. pneumoniae* ∆*dhaM*∆*ptsG*∆*glpK* (Fig. 6). Nearly all the glycerol oxidation pathways were blocked in this strain.

### Glucose oxidation provides NADH for 1,3-PDO synthesis in the co-substrates process

Glycerol cannot enter glycolysis to generate energy and NADH in *K. pneumoniae* ∆*dhaM*∆*ptsG*∆*glpK*. Therefore, in the co-substrate cultivation process, energy and NADH were generated from the catabolism of glucose. When NADH generation was limited, the synthesis of 1,3-PDO ceased (Fig. 7). Under microaerobic conditions, the activity of the TCA cycle is at a low level, and both ATP and NADH generation are lower than that under aerobic conditions [31]. Nevertheless, under fully aerobic conditions, the 1,3-PDO synthesis was inhibited (Fig. 8). Stoichiometric analysis showed that microaerobic conditions are more favourable for 1,3-PDO production than anaerobic or aerobic conditions [21]. It had been reported that low oxygen levels can lead to higher glycerol dehydratase activity, creating an environment favourable for 1,3-PDO formation [4]. Therefore, the strain *K. pneumoniae* ∆*dhaM*∆*ptsG*∆*glpK* was a promising 1,3-PDO-producing strain cultured under microaerobic conditions.

In the fed-batch fermentation, the synthesis of 58.7 g/L of 1,3-PDO consumed 72.21 g/L of glucose (Fig. 9). The glucose consumed was higher than that in shake flasks (Fig. 7). The excess glucose consumed in the process was due to the synthesis of by-products. The synthesis of 2,3-BDO, acetoin, and ethanol from pyruvate all consumed NADH. In addition to competing with 1,3-PDO synthesis for NADH, these by-products enhanced the burden of the downstream process. However, these by-products cannot be eliminated by straightforward methods. The production of 1,3-PDO decreased by blocking the 2,3-BDO and acetate pathway (Fig. S1). It has been reported that blocking the 2,3-BDO pathway will trigger heavy carbon-metabolic traffic at the pyruvate node that reduces glycerol assimilation and 1,3-PDO production [32]. Ways to reduce these by-products but not affecting the 1,3-PDO synthesis needed to be explored.

### The synthesis of 3-HP affects the yield of 1,3-PDO

1,3-PDO and 3-HP were both the final products of the glycerol reduction pathway. In both batch and fed-batch fermentations, the yield of 1,3-PDO was consistently maintained at approximately 0.93 mol/mol glycerol. Theoretically, eliminating the synthesis of 3-HP can achieve a 100% conversion of glycerol to 1,3-PDO.

It has been reported that gamma-aminobutyraldehyde dehydrogenase (encoded by *ydcW*), gamma-glutamyl-gamma-aminobutyraldehyde dehydrogenase (encoded by *puuC*), aldehyde dehydrogenase (encoded by *aldH*), and aldehyde dehydrogenase A (encoded by *aldA*) catalyse the conversion of 3-HPA to 3-HP [33–35]. These genes were knocked out individually, and engineered strains were cultured in shake flasks. The results show that the deletion of genes *ydcW*, *puuC*, or *aldA* does not affect the synthesis of 3-HP, while the deletion of *aldH* leads to restricted growth of the strain (data not shown). These results indicated that there were unidentified enzymes that catalyse the conversion of 3-HPA to 3-HP in *K. pneumoniae*.

Besides 3-HP, by-products of the process include acetate, 2,3-BDO and acetoin. However, these by-products can not be eliminated by straightforward methods. Blocking 2,3-BDO and acetate pathway will trigger heavy carbon-metabolic traffic at the pyruvate node. We are seeking suitable ways to reduce the production of these by-products without affecting 1,3-PDO synthesis.

### *K. pneumoniae* ∆*dhaM*∆*ptsG*∆*glpK* is an efficient strain for 1,3-PDO production with glucose and glycerol as co-substrates

A variety of microorganisms have been reported to be 1,3-PDO producers, including *Klebsiella* [26], *Clostridium* [36], *Enterobacter* [37], *Citrobacter* [38] and *Lactobacillus* [39]. Table 4 shows 1,3-PDO production by bacteria using glucose and glycerol as co-substrates [23, 24, 40].

**Table 4:** Comparison of 1,3-PDO production in various bacterial strains using glucose and glycerol as co-substrates

Strains	VB_12_ Addition	Titer (g/L)	Yield (mol/mol glycerol)	Yield (mol/mol substrate)	Productivity (g/L/h)	References
*C. diolis* DSM 15,410	No	14.7	0.86	0.69	1.1	[23]
*L. brevis* N1E9.3.3	Yes	18.6	1.10		0.78	[46]
*L. reuteri* CH53	No	68.32			1.27	[22]
*L. reuteri* DSM 20,016	No	52.3	0.85	0.49	1.09	[40]
*L. reuteri* ATCC 55,730	No	65.3	0.81		1.2	[42]
*L. diolivorans* DSM 14,421	Yes	85.4		0.57	0.85	[39]
*L. panis* PM1	No	8.2	0.86	0.59	0.41	[47]
*E. coli* JA03	Yes	13.5	0.64	0.41	0.22	[48]
*E. coli* Rosetta	No	41.6	0.67	0.59	0.69	[43]
*E. coli* NSK015	Yes	36.8	0.99	0.61	0.29	[5]
*E. coli* K12	Yes	80.0	0.99	0.66	1.67	[49]
*K. pneumoniae*	No	81.2	0.53	0.48	1.13	[24]
*K. pneumoniae*	No	3.1	0.59	0.55		[44]
*K. pneumoniae*	No	50.3	0.94	0.64	2.09	This work (Batch)
*K. pneumoniae*	No	58.7	0.93	0.63	0.84	This work (Fed batch)

*Clostridium* species, which produce 1,3-PDO under anaerobic conditions, show good performance under a co-substrate system compared to using glycerol as a sole substrate. The yield of 1,3-PDO from glycerol increased from 0.67 mol/mol to 0.79–0.86 mol/mol in *C. diolis*. However, 1,3-PDO production by *Clostridium* faces challenges, including lower biomass efficiency, long seed culture, and genetic manipulation efficiency [23]. Glycerol cannot be used as the sole carbon source for *Lactobacilli* spp. growth due to the lack of dihydroxyacetone kinase, so other carbon sources are required [41]. Using *L. reuteri* as the producer, at a molar ratio of glucose/ glycerol 1.5:1, the conversion of glycerol to 1,3-PDO reached 0.97 mol/ mol, with a final titer of 65.3 g/L. However, lactate produced in the process reached 106.5 g/L, which was a significant burden for the separation of 1,3-PDO [42].

*E. coli* was used for 1,3-PDO production after constructing an artificial 1,3-PDO synthesis pathway. Co-substrate of glycerol and glucose was employed for 1,3-PDO production by an engineered *E. coli*. 36.8 g/L of 1,3-PDO was produced, with a yield of 0.99 mol/ mol glycerol [5]. However, *E. coli* does not have an endogenous vitamin B_12_ synthesis pathway, and vitamin B_12_ had to be supplied in the process. Using a vitamin B_12_-independent glycerol dehydratase, 41.65 g/L of 1,3-PDO was produced by the engineered *E. coli* strain with glucose as the co-substrate. The 1,3-PDO productivity was 0.69 g/L/h, and the yield was 0.67 mol/mol glycerol [43].

Several studies have investigated 1,3-PDO production by *K. pneumoniae* using glucose and glycerol as co-substrates. In one study, the yield of glycerol to 1,3-PDO increased 11% after 1 g/L of glucose was added to the medium [44]. Using a CCR-deficient *K. pneumoniae* strain, the yield of 1,3-PDO from glycerol reached 0.53 mol/mol with a final titer of 81.2 g/L [24]. To increase the yield of 1,3-PDO, *glpK*, *dhaD* and *glpD* were deleted to block the glycerol oxidation pathway. However, the activities of glycerol dehydratase and 1,3-PDO oxidoreductase were significantly reduced in these strains. Thus, an exogenous plasmid was used to express these two enzymes to maintain the 1,3-PDO production [25].

1,3-PDO is a bulk chemical, and the cost of feedstock accounts for a significant portion of the overall production cost. The conversion ratio of glycerol to 1,3-PDO reached 0.629 mol/mol by an engineered *K. pneumoniae* CGMCC 1.6366 with glycerol as the substrate [45]. In this study, *glpK* and *dhaM* are respectively knocked out to block the glycerol oxidation pathways, while preserving the activities of key enzymes of the *dha* pathway. Knocking out *ptsG* was used to alleviate CCR.

In batch fermentation under microaerobic conditions, 60.5 g/L glucose and 64.81 g/L glycerol were consumed after 20 h of cultivation of *K. pneumoniae* ∆*dhaM*∆*ptsG*∆*glpK*, 50.25 g/L 1,3-PDO was produced. The yield of 1,3-PDO reached 0.94 mol/mol glycerol and 0.64 mol/mol carbon source, with a productivity of 2.09 g/L/h. 58.6 g/L of 1,3-PDO was produced in a fed-batch fermentation, with a yield of glycerol to 1,3-PDO of 0.93 mol/mol. 0.5 mol glucose per 1 mol 1,3-PDO was consumed in the process.

## Conclusion

The large subunit of dihydroxyacetone kinase Ⅱ and glycerol kinase were inactivated in the CCR-deficient strain *K. pneumoniae* ∆*ptsG*. Nearly all the glycerol was derived for 1,3-PDO synthesis. NADH and energy were provided by the catabolism of glucose. *K. pneumoniae* ∆*dhaM*∆*ptsG*∆*glpK* proved to be a high yield and high titer producer of 1,3-PDO with glucose and glycerol as co-substrates. Furthermore, the ratio of glucose to glycerol concentration was optimized in the shake flask, 0.5:1 mol/mol was the optimal value for 1,3-PDO production, with a yield of 0.95 mol/mol glycerol. Batch fermentation in bioreactor experiments demonstrated both anaerobic and high oxygen supplement conditions were unfavourable for the production of 1,3-PDO. In fed-batch fermentations, the maximum production of 1,3-PDO is 58.6 g/L with a yield of 0.931 mol/mol glycerol. The high yield demonstrates the great potential of *K. pneumoniae* ∆*dhaM*∆*ptsG*∆*glpK* to produce 1,3-PDO using glucose and glycerol as substrates.

### Methods

### Strains and plasmids

The bacterial strains and plasmids used in this study are listed in Table 5. The primers used in this study are listed in Table S1. *K. pneumoniae* CGMCC 1.6366 was used as the starting strain in this study. *E. coli* DH5α was used as the host strain for plasmid replication. The suicide plasmid pkpsacB was used as the knock-out vector.

**Table 5:** Strains and plasmids

Strains or plasmids	Description	Sources
*E. coli* DH5α	Host for plasmid	Lab stock
*K. pneumoniae* CGMCC 1.6366	TUAC01 wild type	[26]
Δ*dhaM*	Δ*dhaM*	This work
Δ*dhaM*Δ*crr*	Δ*dhaM*, Δ*crr*	This work
Δ*dhaK1*Δ*ptsG*	Δ*dhaK1*, Δ*ptsG*	This work
Δ*dhaK*Δ*ptsG*	Δ*dhaK*, Δ*ptsG*	This work
Δ*dhaL*Δ*ptsG*	Δ*dhaL*, Δ*ptsG*	This work
Δ*dhaM*Δ*ptsG*	Δ*dhaM*, Δ*ptsG*	This work
Δ*dhaM*Δ*ptsG*Δ*puuC*	Δ*dhaM*, Δ*ptsG*, Δ*puuC*	This work
Δ*dhaM*Δ*ptsG*Δ*ydcW*	Δ*dhaM*, Δ*ptsG*, Δ*ydcW*	This work
Δ*dhaM*Δ*ptsG*Δ*aldA*	Δ*dhaM*, Δ*ptsG*, Δ*aldA*	This work
Δ*dhaM*Δ*ptsG*Δ*aldH*	Δ*dhaM*, Δ*ptsG*, Δ*aldH*	This work
Δ*dhaM*Δ*ptsG*Δ*glpK*	Δ*dhaM*, Δ*ptsG*, Δ*glpK*	This work
Δ*dhaM*Δ*ptsG*Δ*dhaD*	Δ*dhaM*, Δ*ptsG*, Δ*dhaD*	This work
Δ*dhaM*Δ*ptsG*Δ*gldA*	Δ*dhaM*, Δ*ptsG*, Δ*gldA*	This work
Δ*dhaD*Δ*gldA*Δ*ptsG*	Δ*dhaD*, Δ*gldA*, Δ*ptsG*	This work
Δ*dhaM*Δ*ptsG*Δ*gldA*Δ*glpK*	Δ*dhaM*, Δ*ptsG*, Δ*gldA*, Δ*glpK*	This work
Δ*dhaM*Δ*ptsG*Δ*dhaD*Δ*glpK*	Δ*dhaM*, Δ*ptsG*, Δ*dhaD*, Δ*glpK*	This work
Δ*dhaM*Δ*ptsG*Δ*gldA*Δ*dhaD*Δ*glpK*	Δ*dhaM*, Δ*ptsG*, Δ*gldA*, Δ*dhaD*, Δ*glpK*	This work
Plasmids		
pkpsacB	Apr^r^, carries pSC101 ori, Rep101(Ts), and *sacB*	This work
pkpsacB-*dhaK1*-KO	pkpsacB carrying *dhaK1* upstream and downstream regions (ATP-dependent)	This work
pkpsacB-*dhaK*-KO	pkpsacB carrying *dhaK* upstream and downstream regions (PEP-dependent)	This work
pkpsacB-*dhaL*-KO	pkpsacB carrying *dhaL* upstream and downstream regions	This work
pkpsacB-*dhaM*-KO	pkpsacB carrying *dhaM* upstream and downstream regions	This work
pkpsacB-*ptsG*-KO	pkpsacB carrying *ptsG* upstream and downstream regions	This work
pkpsacB-*crr*-KO	pkpsacB carrying *crr* upstream and downstream regions	This work
pkpsacB-*gldA*-KO	pkpsacB carrying *gldA* upstream and downstream regions	This work
pkpsacB-*dhaD*-KO	pkpsacB carrying *dhaD* upstream and downstream regions	This work
pkpsacB-*glpK*-KO	pkpsacB carrying g*lpK* upstream and downstream regions	This work
pkpsacB-*aldA*-KO	pkpsacB carrying *aldA* upstream and downstream regions	This work
pkpsacB-*aldH*-KO	pkpsacB carrying a*ldH* upstream and downstream regions	This work
pkpsacB-*puuC*-KO	pkpsacB carrying *puuC* upstream and downstream regions	This work
pkpsacB-*ydcW*-KO	pkpsacB carrying *ydcW* upstream and downstream regions	This work

### Construction of *K. pneumoniae* mutants

The suicide plasmid pkpsacB was constructed for seamless gene editing in *K. pneumoniae.* pkpsacB contains a counter-selection system and a temperature-sensitive origin of replication. It can replicate below 30 °C, but not at 37 °C. Cells containing this plasmid cannot live in sucrose-containing media due to the presence of the counter-selectable marker *sacB* gene. The target genes on the chromosome (Table 5) were knocked out via a double homologous recombination with pkpsacB. The knockout of *ptsG* as an example. 1000 ~ 1500 bp of the upstream and downstream sequences of the gene were amplified using primer pairs: *ptsG*-up-F/*ptsG*-up-R and *ptsG*-dn-F/*ptsG*-dn-R, respectively. The upstream and downstream fragments were ligated into the pkpsacB vector to construct the plasmid pkpsacB-*ptsG*-KO using a ClonExpress Ultra One Step Cloning Kit (Vazyme, China). pkpsacB-ptsG-KO was introduced into *K. pneumoniae* by electroporation and cultivated at 30 °C. After 12 h of cultivation in an apramycin-added liquid medium, the cultivation temperature was increased to 37 °C. pkpsacB-ptsG-KO that contains regions of homology was integrated into the chromosome of *K. pneumoniae* via an allelic exchange under antibiotic and temperature selection. A second allelic exchange was then performed to remove the plasmid backbone by growing the cells on antibiotic-free medium. After several generations of cultivation, colonies were purified by streaking on the TYS6 plate and confirmed by PCR and DNA sequencing.

### Fermentation conditions

#### Microorganisms

*K. pneumoniae* and *E. coli* were grown in Luria–Bertani (LB) medium at 37 °C. TYS6 (10 g/L of Tryptone, 5 g/L of Yeast extract and 10% (W/V) Sucrose) was used in the selection of the second crossover event. The concentration of apramycin used in the selective medium was 50 µg/mL.

#### Fermentation medium

The fermentation medium contained 4.2 g/L (NH_4_)_2_SO_4_, 2 g/L yeast extract, 7 g/L K_2_HPO_4_·3H_2_O, 2.5 g/L KH_2_PO4, 0.25 g/L MgSO_4_, 0.02 g/L FeSO_4_·7H_2_O and 1 mL of trace element solution [7]. Different concentrations of carbon sources (glucose and glycerol) were added to the fermentation medium.

Shake-flasks experiments.

In shake-flask experiments, seed cultures at a cell density of 0.1 OD units were inoculated into 50 mL fermentation medium containing 18 g/L of glucose and 18 g/L of glycerol in a 250 mL flask at 37 °C, 150 rpm for 15 h. The ratio of glucose to glycerol in the medium, containing 13 g/L of glycerol with glucose of different concentrations, was optimised in shake-flask experiments. 11 experimental groups were done; the concentrations of glucose and glycerol are listed in Table 2. After 24 h of cultivation, the metabolites in the broth were detected.

#### Batch fermentation in bioreactors

Bioreactor fermentation was performed in 5 L tank bioreactors (BIOSTAT-A plus, Sartorius) with a working volume of 3 L. *K. pneumoniae* ∆*dhaM*∆*ptsG*∆*glpK* was cultivated in 50 mL flasks at 37 °C, 150 rpm for 15 h as a seed. Then the seed was inoculated into bioreactors. Aeration rate of 0, 2–4 L/min and agitation speeds of 200 or 400 rpm were used, according to the experimental design. The culture pH was automatically controlled at 7.0 by 10 mol/L NaOH.

#### Fed-batch fermentation in bioreactors

In fed-batch experiments, the initial medium contained 85 g/L of glucose. Glycerol was fed after 4 h of cultivation at a final concentration of 10 g/L. *K. pneumoniae* ∆*dhaM*∆*ptsG*∆*glpK* was inoculated into the medium with a cell density of 0.1 OD units. Temperature, aeration rate, agitation and pH were maintained at 37 °C, 2 L/min, 200 rpm and 7.0 during fermentation, respectively. Glucose was fed when its concentration dropped below 5 g/L. 5 mL of broth was sampled every 4–5 h for analyses.

### Analytical methods

Biomass concentration was determined by measuring the optical density at 600 nm with a spectrophotometer. Compounds, including glucose, glycerol, acetate, succinate and ethanol in the broth were quantified by high-performance liquid chromatography (HPLC) and gas chromatography system (GC) as previously described [10]. Statistical significance was assessed using a t-test with GraphPad Prism (10.1.2). Significant differences between means were defined as *P* < 0.05 level.

## Supplementary Information

Below is the link to the electronic supplementary material.

Supplementary Material 1

## Data Availability

The datasets generated and/or analysed during the current study are available from the corresponding author upon reasonable request.
