# Supplementary material for: 1,3-propanediol production by Klebsiella pneumoniae ∆dhaM∆ptsG∆glpK using glucose and glycerol as co-substrates
Source: Microb Cell Fact. 2025 Dec 15;25:13. doi: 10.1186/s12934-025-02896-6 (PMC12821955; doi:10.1186/s12934-025-02896-6)

1,3-Propanediol production by *Klebsiella pneumoniae* ∆*dhaM*∆*ptsG*∆*glpK* using glucose and glycerol as co-substrates

Shaoqi Sun 1,3, Weiyan Jiang 1,3, Yaoyu Cai 1,3, Wenqi Wang 1,3, Xinjie Bian 1,3, Taiyu Liu 1, Marina Tišma 2, Dexin Wang1*, and Jian Hao 1,3*

1. Lab of Biorefinery, Shanghai Advanced Research Institute, Chinese Academy of Sciences, No. 99 Haike Road, Pudong, Shanghai 201210, People’s Republic of China

2. Josip Juraj Strossmayer University of Osijek, Faculty of Food Technology Osijek, Franje Kuhača 18, Osijek, HR-31000, Croatia

3. University of Chinese Academy of Sciences, Beijing, 100049, PR China

*Corresponding author: Dexin Wang, Jian Hao

Email: wangdx@sari.ac.cn; haoj@sari.ac.cn Tel.: +86 21 20325163

**Table S1** Primers used in this study

| Primer | Sequence |
| --- | --- |
| dhaM-up-F | TCCTGTTTGAAGATGGCAAGCTTCTCCATCACCCTGTTGAAAG |
| dhaM-up-R | AATGGTCGACGGATTCGATCGCTTCCA |
| dhaM-dn-F | GATCGAATCCGTCGACCATTATTATCGCTGACAACA |
| dhaM-dn-R | GGTACCCGGGGATCCTCTAGGATGTCCGCACTGTTTTGCG |
| ptsG-up-F | TCCTGTTTGAAGATGGCAAGCCTGCTGCGCTGGGAACA |
| ptsG-up-R | TAACTGTTACGGCCGACCTTCTGCAGGTTAGCA |
| ptsG-dn-F | AAGGTCGGCCGTAACAGTTAAGACGTAGTATCGG |
| ptsG-dn-R | GGTACCCGGGGATCCTCTAGGCTGTGACGGGACGGATGG |
| crr-up-F | TCCTGTTTGAAGATGGCAAGCACACCTCCATCATGGCGC |
| crr-up-R | TCTTGATGCGCCCATGATCTTCTCCTAAGCAGTAA |
| crr-dn-F | AGATCATGGGCGCATCAAGAAGTAATATCTGCTGC |
| crr-dn-R | GGTACCCGGGGATCCTCTAGGCATAGGACGAATCATAATCAGAGC |
| glpK-up-F | TCCTGTTTGAAGATGGCAAGCGATCGCGTCGACGTCGT |
| glpK-up-R | AATCTTACGCTTCGTCGGTCGGTCATAGTCGGTGTCCC |
| glpK-dn-F | GACCGACGAAGCGTAAGATTGTTGA |
| glpK-dn-R | GGTACCCGGGGATCCTCTAGCCCAGGCTTTGATCTGAATAATG |
| puuC-up-F | TCCTGTTTGAAGATGGCAAGCAAAGACGATTAAACCCCAAGG |
| puuC-up-R | ATATTGCGCCCCATCTGGATTGCCCTGGA |
| puuC-dn-F | ATCCAGATGGGGCGCAATATTCGCCGTT |
| puuC-dn-R | GGTACCCGGGGATCCTCTAGGTCCCATCAGGTTGAGGTTCAG |
| aldH-up-F | TCCTGTTTGAAGATGGCAAGGCCGATTACCGATATCGCCTTG |
| aldH-up-R | ACCAGTGAAGCTTGCCATCTTCAAACAGGAGG |
| aldH-dn-F | GGCTCTTGTATCTATCAGTGGAGTGCACCATAATCGGCATT |
| aldH-dn-R | GGTACCCGGGGATCCTCTAGCCGACACCACGCTTATCAGC |
| ydcW-up-F | TCCTGTTTGAAGATGGCAAGGCCTATATTAAGGTCATAAACAATACGG |
| ydcW-up-R | CAACACAACCATGGCTAACGGATTGACCCA |
| ydcW-dn-F | CGTTAGCCATGGTTGTGTTGCATAGCATTATCCT |
| ydcW-dn-R | GGTACCCGGGGATCCTCTAGGCTTCCTGGTCTTCGTCTACATCT |
| aldA-up-F | TCCTGTTTGAAGATGGCAAGTCAAACCATTCGATGGCGC |
| aldA-up-R | AGATAGACCGTTGAACGGGTGCTGTCATGG |
| aldA-dn-F | ACCCGTTCAACGGTCTATCTGCAGGCCTGAGG |
| aldA-dn-R | GGTACCCGGGGATCCTCTAGGTGGATCAGGCCTGGAATATAGC |
| dhaD-up-F | TCCTGTTTGAAGATGGCAAGGCTCGCTTTCATTGTGGATGG |
| dhaD-up-R | TGCTGCTGTCCGATCTGTTAGGCCAGCAGTG |
| dhaD-dn-F | TAACAGATCGGACAGCAGCATCAGGACCCTG |
| dhaD-dn-R | GGTACCCGGGGATCCTCTAGGAACTCCATTTCATCGTCCTGC |
| gldA-up-F | TCCTGTTTGAAGATGGCAAGGTGCAGCAATGGCTCTCACTG |
| gldA-up-R | GAAATGGATCGCTTCCTGCAGGAATGGGA |
| gldA-dn-F | TGCAGGAAGCGATCCATTTCGATTCCTCCTCG |
| gldA-dn-R | GGTACCCGGGGATCCTCTAGCTGTTCGAGGCAATCCGTCA |
| dhaK-up-F | TCCTGTTTGAAGATGGCAAGGGTGTAGATCACCGACAGCGC |
| dhaK-up-R | GTGCCATCTTCAACACGGTTAATCAGCT |
| dhaK-dn-F | AACCGTGTTGAAGATGGCACCTCGCTGGATATGGC |
| dhaK-dn-R | GGTACCCGGGGATCCTCTAGAGCAACGCGCTGCCGATA |
| dhaL-up-F | TCCTGTTTGAAGATGGCAAGTGCTGATTATCAAAAACTACACCGG |
| dhaL-up-R | AAACATCACTGACGTGGCCAGCCAGTCGACGATTTGCG |
| dhaL-dn-F | TGGCCACGTCAGTGATGTTTATGATG |
| dhaL-dn-R | GGTACCCGGGGATCCTCTAGTTGCGCCGCAGGCGATTC |
| dhaK1-up-F | TCCTGTTTGAAGATGGCAAGGCTGGCCCGCGGGCCAGA |
| dhaK1-up-R | TAAACACCATCGCTACCGCTCGTTGACGAGGCTGGCG |
| dhaK1-dn-F | AGCGGTAGCGATGGTGTTTAAA |
| dhaK1-dn-R | GGTACCCGGGGATCCTCTAGGCAGACAAGTACCCGCAGCG |

**Figure S1:** 1,3-PDO production and substrate consumption of *pta* or *budB* knocked out strains in flask experiments using glucose and glycerol as co-substrates. Error bars represent standard deviations (n=3).


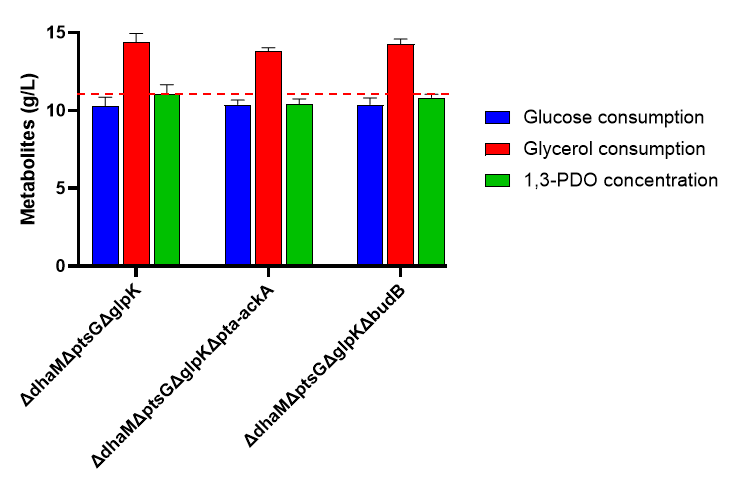

Supplement: Supplementary file 1 — Supplementary Material 1 [file 12934_2025_2896_MOESM1_ESM.docx]
